# Efficacy and safety of neoadjuvant immunotherapy combined with chemoradiotherapy or chemotherapy in esophageal cancer: A systematic review and meta-analysis

**DOI:** 10.3389/fimmu.2023.1117448

**Published:** 2023-01-24

**Authors:** Yunsong Liu, Yongxing Bao, Xu Yang, Shuang Sun, Meng Yuan, Zeliang Ma, Wanting Zhang, Yirui Zhai, Yang Wang, Yu Men, Jianjun Qin, Liyan Xue, Jun Wang, Zhouguang Hui

**Affiliations:** ^1^ Department of Radiation Oncology, National Cancer Center/National Clinical Research Center for Cancer/Cancer Hospital, Chinese Academy of Medical Sciences and Peking Union Medical College, Beijing, China; ^2^ Department of Mathematics and Statistics, Lancaster University, Lancaster, United Kingdom; ^3^ Department of very important person (VIP) Medical Services and Radiation Oncology, National Cancer Center/National Clinical Research Center for Cancer/Cancer Hospital, Chinese Academy of Medical Sciences and Peking Union Medical College, Beijing, China; ^4^ Department of Thoracic Surgery, National Cancer Center/National Clinical Research Center for Cancer/Cancer Hospital, Chinese Academy of Medical Sciences and Peking Union Medical College, Beijing, China; ^5^ Department of Pathology, National Cancer Center/National Clinical Research Center for Cancer/Cancer Hospital, Chinese Academy of Medical Sciences and Peking Union Medical College, Beijing, China; ^6^ Department of Radiotherapy, The Fourth Hospital of Hebei Medical University, Shijiazhuang, China

**Keywords:** efficacy, safety, neoadjuvant, immunotherapy, chemotherapy, chemoradiotherapy, esophageal cancer, meta-analysis

## Abstract

**Background:**

Significant progress has been made in the investigation of neoadjuvant immune-chemoradiotherapy (NICRT) and neoadjuvant immune-chemotherapy (NICT) on the outcomes of esophageal cancer patients. To summarize the current developments, a systematic review and meta-analysis were conducted to evaluate the efficacy and safety of neoadjuvant immunotherapy combined with chemoradiotherapy or chemotherapy.

**Methods:**

A search strategy of prospective studies on esophageal cancer receiving neoadjuvant immunotherapy was predefined to scan PubMed, Embase, Cochrane, and additional major conferences for prospective studies. Efficacy was assessed by pathological complete response (pCR), major pathological response (MPR), and R0 resection rates. Safety was evaluated based on the incidence of grade ≥ 3 treatment-related adverse events (TRAEs), neoadjuvant therapy completion rate, surgical resection rate, and surgical delay rate. Differences between the NICRT and NICT groups were also analyzed.

**Results:**

A total of 38 studies qualified for the analysis. The pooled pCR, MPR, and R0 resection rates were 30, 58, and 99%, respectively. The pCR and MPR in the NICRT vs. NICT group were 38% vs. 28% (p=0.078) and 67% vs. 57% (p=0.181), respectively. The pooled incidence of grade ≥ 3 TRAEs was 24% (NICRT,58%, I^2^ = 61% vs. NICT,18%, I^2^ = 79%; p<0.001). In addition, the pooled neoadjuvant therapy completion and surgical resection rates were 92% and 85%, respectively; the difference was not statistically significant between the NICRT and NICT groups.

**Conclusions:**

Neoadjuvant immunotherapy combined with chemoradiotherapy or chemotherapy is effective and safe in the short term for locally advanced esophageal cancer. However, further randomized trials are needed to confirm which combined model is more favorable.

**Systematic review registration:**

https://www.crd.york.ac.uk/prospero/display_record.php?ID=CRD42021284266, identifier CRD42021284266.

## 1 Introduction

Esophageal cancer is a serious public health threat, ranking seventh in term of incidence and sixth in term of mortality among all cancers (1). Moreover, the overall 5-year survival rate is about 20% as one of the four cancer types with lowest survival (2).

Neoadjuvant chemoradiotherapy following surgery is considered as one of the standard treatments of choice for local advanced resectable esophageal cancer, with a median overall survival (OS) of 49.4 months, and a pathological complete response (pCR) rate of 29%, as reported in the CROSS trial (3). NEOCRTEC 5010, a phase III randomized controlled trial (RCT), compared neoadjuvant chemoradiation in esophageal squamous cell carcinoma (ESCC) and reported more appealing results with a median OS of 100.1 months and a pCR of 43.2% (4). However, even after neoadjuvant chemoradiotherapy combined with surgery, the 5-year overall survival and progression-free survival (PFS) rates were approximately 47% and 44%, respectively, indicating that a considerable proportion of patients still experience progression of the disease and eventually death (5). In addition, the safety of chemoradiation is a noteworthy problem because some patients cannot tolerate neoadjuvant chemoradiotherapy (4).

Neoadjuvant chemotherapy is an alternative first-line treatment for esophageal cancer, especially in East Asia. A randomized trial, JCOG9907, showed a 5-year OS of 55% in patients with resectable ESCC receiving prior two courses of cisplatin and 5-fluorouracil (6). Perioperative chemotherapy is usually recommended for resectable esophageal adenocarcinoma (EAC). The randomized phase II/III FLOT-4 trial reported a median OS of 50 months for patients receiving the perioperative FLOT regimen, with a pCR of 15% (7). A meta-analysis including three RCTs showed a pCR of < 10% in patients having received neoadjuvant chemotherapy, which was significantly lower than in those who had received neoadjuvant chemoradiotherapy, although survival for neoadjuvant chemotherapy may not be inferior probably due to less toxicity and salvage treatment (8). Briefly, neoadjuvant chemotherapy may achieve acceptable survival rates, but its short-term efficacy is unfavorable for esophageal cancer.

Immune checkpoint inhibitors (ICIs) immunotherapy has been demonstrated to be effective in improving survival and safety in patients with stage IV esophageal cancer (9–11). Neoadjuvant chemoradiation or chemotherapy combined with ICIs for esophageal cancer have been studied in a series of small clinical trials for resectable esophageal cancer (12, 13), showing promising results. Majority of the trials focused on ESCC. A systematic search of the literature and a meta-analysis of multiple clinical trials will help summarize the evidence and provide comprehensive information for clinicians. This meta-analysis aimed to investigate the efficacy and safety of neoadjuvant immunotherapy, and to compare neoadjuvant immunotherapy combined with chemoradiotherapy or chemotherapy for resectable esophageal cancer, therefore providing evidence of neoadjuvant immunotherapy mainly for ESCC. Our main purpose for EAC was to provide a rough reference due to limited available data.

## 2 Methods

We followed the Preferred Reporting Items for Systematic Reviews and Meta-Analyses (PRISMA) guidelines to conduct systematic reviews and meta-analyses (14).

### 2.1 Search strategy

A systematic search was conducted in English on PubMed, Embase, and the Cochrane Library to identify articles on neoadjuvant immunotherapy for esophageal cancer reported before July 14, 2022. Abstracts of several important international conferences, such as ASCO, ESMO, and AACR occurring up to July 14, 2022, were also inspected. Keywords included “esophageal cancer”, “neoadjuvant” and “immunotherapy”. The complete search strategy is available in the Supplementary Material. The protocol is registered in the PROSPERO database (CRD42021284266).

### 2.2 Study selection

The eligibility criteria were as follows: 1) patients with resectable esophageal cancer who received neoadjuvant immunotherapy combined with chemoradiotherapy or chemotherapy, 2) prospective studies with data available on pCR or major pathological response (MPR) rates, and 3) ICIs are currently used in clinical practice or in registered trials.

The exclusion criteria were as follows: 1) the number of patients available for analysis was less than 10, 2) repeated publications, 3) case reports, reviews, and experimental reports and 4) lack of valid data.

### 2.3 Data extraction

The following information was extracted: first author, publication year, clinical trial registry number, intervention model, study phase, article type, neoadjuvant therapy mode, main inclusion criteria, ICI drug and dose, sample size, sex, median/mean age, pathological complete response (pCR), major pathological response (MPR), R0 resection rate, incidence of ≥ grade 3 treatment-related adverse events (TRAEs), neoadjuvant therapy completion rate (NTCR), surgical resection rate, and surgical delay rate. The rates of pCR, MPR, and R0 resection were calculated based on patients who had undergone surgery. pCR was defined as the absence of residual tumor in all resected specimens (i.e., ypT0N0). MPR referred to 10% or less of residual viable cancer cells after neoadjuvant therapy (15, 16). R0 corresponded to microscopic margin-negative resection. The incidence of ≥grade 3 TRAEs, NTCR and surgical resection rate were calculated based on the intention-to-treat population. TRAEs≥grade 3 were counted based on the National Cancer Institute Common Terminology Criteria for Adverse Events (NCI-CTCAE). NTCR was defined as the ratio of patients who successfully completed the entire course of neoadjuvant therapy within the total number of enrolled patients. The surgical resection rate refered to the ratio of patients receiving surgery to those whose surgery was anticipated. Two authors (LYS and BYX) independently reviewed articles and extracted the data from the included trials. Disagreements were resolved through discussion between the two reviewers until a consensus was reached.

### 2.4 Statistical analysis

The random effects model using DerSimonian-Laird method was applied because homogeneity might not be validated under the current scenario. The random effects model considers the case in which the effect size is more variable when compared with a homogeneous population. The pooled results were presented as incidence rates with 95% confidence interval (CI). The pooled proportion was calculated with the formula: logit(P)=ln(P/(1-P)). Between-study heterogeneity was assessed using tau^2^ and I^2^ statistics. A p-value>0.1 and an I^2^<50% indicated that the heterogeneity was acceptable. A comparative analysis was carried out between the neoadjuvant immune-chemoradiotherapy (NICRT) group and the neoadjuvant immune-chemotherapy (NICT) group to explore the respective efficacy and safety, as well as acquire a preliminary comparison. The difference between subgroups was tested using the chi-square test, and statistical significance was set at p < 0.05. Notably, some of the studies were still ongoing; we did not include those without all enrolled patients reaching the planned surgery time when pooling the neoadjuvant therapy completion rate, incidence of ≥grade 3 TRAEs, surgical resection rate, and surgical delay rate because of the incomplete incidence of events.

Exploratory subgroup analysis was performed to evaluate the source of heterogeneity and the association between clinical factors and outcomes. Sensitivity analysis was conducted using leave-one-out method by successively omitting each included study, to examine whether the pooled results were affected by a single study. Egger’s and Begg’s tests were performed to evaluate the publication bias of outcomes with no fewer than 10 available studies. Statistical analyses were performed using the R 4.1.0 program.

### 2.5 Risk of bias and certainty assessment

Since the studies were non-randomized, the risk of bias was assessed following the methodological index for non-randomized studies (MINORS) index (17) which considers the following aspects: a clearly stated aim, inclusion of consecutive patients, prospective collection of data, endpoints appropriate to the aim of the study, unbiased assessment of the study endpoint, follow-up period appropriate to the aim of the study, loss to follow-up of less than 5%, prospective calculation of the study size, adequate control group, contemporary groups, baseline equivalence of groups, and adequate statistical analyses. Certainty of evidence was evaluated using GRADE approach (18), which defines the certainty of evidence into 4 categories: high certainty, moderate certainty, low certainty and very low certainty. The assessments were conducted independently by two researchers, and in case of disagreement, a decision was made through discussion until a consensus was reached.

## 3 Results

### 3.1 Identification of studies

**Figure 1:**
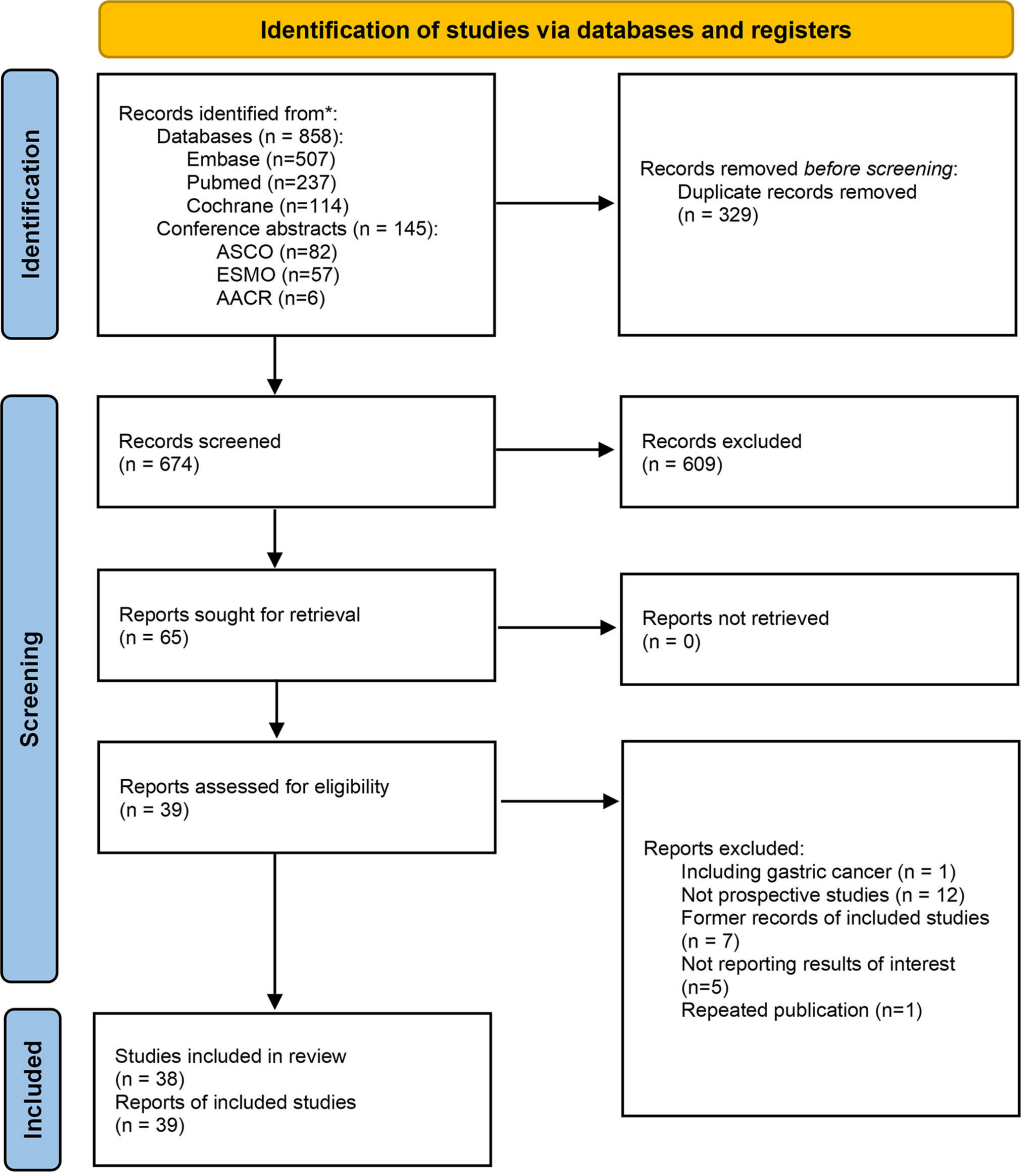
Flow chart of the selection process. *The literature search were performed up to July 14, 2022.

The search identified 1003 records based on the discussed search strategy. First, 329 duplicates were eliminated, and 609 were removed after reviewing the titles and abstracts. 39 records, referring to 38 studies (two records reported different arms of the same trial), were finally selected after full-text review (12, 13, 19–56). The details of the selection process are shown in **Figure 1**. The characteristics of the included studies are summarized in **Table 1**. The main results of each study are presented in **Table 2**. A total of 1252 patients were included in these 38 prospective studies, with 32 single-arm, 3 dual-arm, 1 multi-arm trial and 2 observational cohort studies. 967 patients completed surgery, and reasons for not receiving surgery mainly include not reaching surgery time in ongoing trials, disease progression, toxicity, and patients’ choice. The included trials were similar in terms of the inclusion criteria, age, and sex composition. The median/mean age ranged from 58.3 to 68 years. The proportion of male ranged from 73% to 96% (median 87%). 32 studies enrolled only ESCC (916 patients), 3 studies enrolled only EAC (116 patients) and 3 studies didn’t restrain participants’ histological types (220 patients).

**Table 1:** Characteristics of included studies.

Authors& Publiation year	Registration number	Intervention model	Study phase	Type of publication	Group	Modality of neoadjuvant therapy	Chemotherapy regimen	Radiotherapy regimen	Main inclusion criteria	ICI	Dose of ICI	Cycles of ICI
Cower, D., et al. (19), 2022	NCT02962063	single-arm	II	abstract	NICRT	induced chemotherapy plus concurrent NICRT	induced mFOLFOX; concurrent 5-FU/CAP+OX or paclitaxel/carboplatin	50.4Gy	resectable cTxN+ or T3-4Nx, M0 esophageal/GEJ adenocarcinoma	durvalumab	1500mg iv Q4w	2
Jiang, N., et al. (23), 2022	ChiCTR2100045104	single-arm	II	abstract	NICRT	concurrent NICRT	paclitaxel, carboplatin	30Gy/12f	resectable cT3-4aN0 or cT1-4aN+, M0 ESCC	toripalimab	240mg iv Q3w	2
Kelly, R. J., et al. (32), 2022	NCT03044613	dual-arm	II	abstract	NICRT	cohort A & original cohort B: induced immunotherapy plus concurrent NICRT; amended cohort B: induced immunotherapy plus concurrent CRT	paclitaxel, carboplatin	41.1Gy/23f	stage II-III esophageal/GEJ cancer	cohort A: nivolumab; cohort B: nivolumab+relatlimab	cohort A: 240mg Q2w; cohort B: 240mg Q2w(nivolumab)+80mg Q2w(relatlimab)	cohort A & original cohort B:5; amended cohort B: 2
Lee, S., et al. (33), 2019	NCT02844075	single-arm	II	abstract	NICRT	concurrent NICRT	paclitaxel, carboplatin	44.1Gy/21f	stage IB-III ESCC	pembrolizumab	200 mg iv Q3w	2
Li, C., et al. (12), 2021	NCT03792347	single-arm	IB	article	NICRT	concurrent NICRT	paclitaxel, carboplatin	41.4Gy/23f	resectable cT2-T4aNxM0 ESCC	pembrolizumab	2 mg/kg iv Q3w	2
Manish A. Shah, et al. (39), 2021	NCT02998268	dual-arm	II	abstract	NICRT	cohort A: induced chemotherapy plus concurrent NICRT; cohort B: induced immuno-chemotherapy plus concurrent NICRT	paclitaxel, carboplatin	41.4Gy/23f	resectable cT3-4Nx or T2N1, M0 esophageal/GEJ adenocarcinoma	pembrolizumab	200 mg iv Q3w	NA
Uboha, NV., et al. (25), 2022	NCT03490292	Single-arm	I/II	abstract	NICRT	concurrent NICRT	paclitaxel, carboplatin	41.4Gy/23f	potentially curable cT1N1 or cT2-3N0-2, M0 esophageal/GEJ SCC/AC	avelumab	10mg/kg iv Q2w	3
Qi, W.X., et al. (41), 2020	NA	single-arm	IB	abstract	NICRT	concurrent NICRT	paclitaxel, carboplatin	41.4Gy/23f	local advanced resectable ESCC	pembrolizumab	2 mg/kg iv Q3w	2
van Den Ende, T., et al. (13), 2021	NCT03087864	single-arm	II	article	NICRT	concurrent NICRT	paclitaxel, carboplatin	41.4Gy/23f	resectable <T4b, NxM0 esophageal/GEJ adenocarcinoma	atezolizumab	1200 mg iv Q3w	2
Xu, X., et al. (28), 2022	NCT04437212	single-arm	II	abstract	NICRT	concurrent NICRT	paclitaxel, cisplatin	41.4Gy/23f	resectable cT1-4aN1-2 or T3-4aN0,MO ESCC	toripalimab	240mg iv Q3w	2
Duan, H., et al. (29), 2021	NA	single-arm	NA	article	NICT	concurrent NICT	docetaxel, paclitaxel nedaplatin		resectable cT2-xNxM0 ESCC	sintilimab	200 mg iv Q3w	3
Gao, L., et al. (20), 2022	ChiCTR2100052784	single-arm	II	article	NICT	concurrent NICT	docetaxel, cisplatin		resectable cT3-4Nx or cTxN+, M0 ESCC	toripalimab	240 mg iv Q3w	2
Gu, Y., et al. (30), 2020	NCT03946969	single-arm	IB/II	abstract	NICT	concurrent NICT	paclitaxel, carboplatin, S1		resectable cT1b-T3NxM0 ESCC	sintilimab	200 mg iv Q3w	2
Guo, J., et al. (21), 2022	ChiCTR2000040345	single-arm	II	abstract	NICT	concurrent NICT	paclitaxel, nedaplatin		resectable cT2-4N1-3M0 ESCC	sintilimab	200mg iv Q3w	2
He, W., et al. (31), 2022	NCT04177797	single-arm	II	article	NICT	concurrent NICT	paclitaxel, carboplatin		resectable cT3N1-3M0 ESCC	toripalimab	240 mg iv Q3w	2
Jiang, B., et al. (22), 2022	NA	single-arm	II	abstract	NICT	concurrent NICT	paclitaxel, cisplatin		resectable stage III ESCC	camrelizumab/sintilimab/tislelizumab	200mg iv Q3w	2
Li, K., et al. (34), 2020	ChiCTR2000035237	single-arm	pilot study	abstract	NICT	concurrent NICT	paclitaxel, carboplatin		resectable cT2-T4N0-N2M0 ESCC	toripalimab	240 mg iv Q3w	2~3
Li, Z., et al. (24), 2022	NA	single-arm	II	abstract	NICT	concurrent NICT	paclitaxel, cisplatin		locally advanced ESCC	sintilimab	200mg iv Q3w	2~4
Liu, D., et al. (35), 2021	ChiCTR1900025318	single-arm	II	abstract	NICT	concurrent NICT	paclitaxel, cisplatin		resectable cT1-cT2N+ or cT3-cT4aNx ESCC	toripalimab	240 mg iv Q3w	2
Liu, J.; Li, J., et al. (36), 2022	NCT04225364	single-arm	II	article	NICT	concurrent NICT	paclitaxel, cisplatin		resectable stage II-IVA ESCC	camrelizumab	200 mg iv Q3w	2
Liu, J.; Yang, Y., et al. (37), 2022	ChiCTR1900026240	single-arm	II	article	NICT	concurrent NICT	paclitaxel, carboplatin		resectable ESCC, staged as T1b-4a, N2-3 (≥ 3 stations), M0 or M1 lymph node metastasis (confined to the supraclavicular lymph nodes)	camrelizumab	200 mg iv Q3w	2
Ma, J., et al. (38), 2021	ChiCTR2000033761	single-arm	II	abstract	NICT	concurrent NICT	paclitaxel, nedaplatin		resectable IIA-IIIB ESCC	camrelizumab	200 mg iv Q3w	2~4
Shang, X., et al. (42), 2021	NCT 04389177	single-arm	II	abstract	NICT	concurrent NICT	paclitaxel, cisplatin		potentially resectable cT3N1 or cT1-3N2, M0 ESCC	pembrolizumab	200 mg iv Q3w	3
Shen, D., et al. (43), 2021	NA	single-arm	pilot study	article	NICT	concurrent NICT	paclitaxel, carboplatin		potentially curable cT1N1-3 or cT2-4aNx, M0 ESCC	nivolumab/pembrolizumab/camrelizumab	nivolumab 3mg/kg iv Q3w, pembrolizumab 2mg/kg iv Q3w, camrelizumab 200mg iv Q3w	2
Wang, F., et al. (55), 2021	NCT03917966	single-arm	II	abstract	NICT	induced immunotherapy plus concurrent NICT	docetaxel, nedaplatin		stage II-IVA ESCC	camrelizumab	200 mg iv Q3w	1+2
Wang, W., et al. (26), 2022	NA	single-arm	II	abstract	NICT	concurrent NICT	docetaxel, cisplatin/carboplatin		resectable stage IIB-IVA ESCC	pembrolizumab	200mg iv Q3w	4
Wang, Z., et al. (44), 2021	ChiCTR1900023880	single-arm	IB	abstract	NICT	concurrent NICT	paclitaxel, nedaplatin, apatinib		resectable cT1N2 or T2-3N0-2 ESCC	camrelizumab	200 mg iv Q2w	2~4
Xing, W., et al. (45), 2021	NCT03985670	dual-arm	II	article	NICT	concurrent NICT	paclitaxel, cisplatin		resectable stage II-IVA ESCC	toripalimab	240 mg iv Q3w	2
Xu, W., et al. (56), 2022	NCT04506138	single-arm	II	abstract	NICT	concurrent NICT	paclitaxel, carboplatin		resectable cT2-4aNx or cT1-3N+, M0 ESCC	camrelizumab	200 mg iv Q3w	2
Yamamoto, S.; Matsuda, S., et al. (40, 46), 2021&2022	NCT03914443	multi-arm	I	abstract	NICT	cohort A&C: concurrent NICT; cohort B&D: induced immunotherapy plus concurrent NICT	cohort A&B: cisplatin, 5-FU; cohort C&D: cisplatin, 5-FU, docetaxel		resectable cT1N1-3 or cT2-3Nx, M0 ESCC	nivolumab	cohort A&C: 360mg iv Q3w; cohort B&D: 240 mg lead-in followed by 360 mg iv Q3w	cohort A: 2; cohrot B&C: 3; cohort D: 4
Yan, X., et al. (47), 2021	ChiCTR2000037488	single-arm	II	abstract	NICT	concurrent NICT	paclitaxel, carboplatin		resectable stage II-IVA ESCC	tislelizumab	200 mg iv Q3w	3
Yang, P., et al. (48), 2021	ChiCTR2100051903	single-arm	pilot study	article	NICT	concurrent NICT	paclitaxel, carboplatin		potentially curable cT1N1-3 or cT2-4aNx, M0 ESCC	camrelizumab	200 mg iv Q3w	2
Yang, W., et al. (49), 2021	ChiCTR2000028900	single-arm	pilot study	article	NICT	concurrent NICT	paclitaxel, carboplatin		resectable stage II-III ESCC	camrelizumab	200 mg iv Q3w	2
Zhang, G., et al. (50), 2020	ChiCTR1900027160	NA	observational study	abstract	NICT	concurrent NICT	paclitaxel, S1		resectable stage I-III ESCC	toripalimab	NA	2~4
Zhang, X (51)., 2021	ChiCTR2000029807	single-arm	II	abstract	NICT	concurrent NICT	paclitaxel, S1		resectable stage II-III thoracic ESCC	camrelizumab	200mg iv Q3w	3
Zhang, Y., et al. (52), 2022	ChiCTR2000039170	NA	Observational study	abstract	NICT	NA	primarily paclitaxel and nedaplatin		resectable EC	camrelizumab	NA	NA
Zhang, Z.; Hong, Z., et al. (53), 2021	ChiCTR2100045659	single-arm	II	article	NICT	concurrent NICT	paclitaxel, cisplatin		cT3-4aN0-3 or cT1-2N1-3, M0 ESCC	sintilimab	200 mg iv Q3w	2
Zhang, Z.; Ye, J., et al. (54), 2021	ChiCTR1900026593	single-arm	II	abstract	NICT	concurrent NICT	paclitaxel, carboplatin		resectable stage II-IVA ESCC	sintilimab	200 mg iv Q3w	2

ICI, immune checkpoint inhibitors; NICRT, neoadjuvant immune-chemoradiotherapy; NICT, neoadjuvant immune-chemotherapy; GEJ, gastroesophageal junction; EC, esophageal cancer; ESCC, esophageal squamous cell carcinoma; NA, not available; CAP, capecitabine; OX, oxaliplatin.

**Table 2:** Results of included studies.

Authors	Sample size	Male	Median/Mean age	pCR	MPR	R0 resection rate	Incidence of ≥ grade 3 TRAEs	Neoadjuvant therapy completion rate	Surgical resection rate	Surgical delay rate	Whether all patients reached surgery time
Cower, D., et al. (19)	36	NA	NA	8/33	22/33	NA	NA	NA	33/36	NA	yes
Jiang, N., et al. (23)	23	NA	NA	11/20	16/20	NA	16/23	21/23	20/23	NA	yes
Kelly, R. J., et al. (32)	32	81%	65	9/31	16/31	NA	NA	NA	31/32	NA	not mentioned
Lee, S., et al. (33)	28	89%	60	6/26	NA	25/26	NA	NA	26/28	NA	yes
Li, C., et al. (12)	20	95%	62	10/18	16/18	17/18	13/20	19/20	18/20	0	yes
Manish A. Shah, et al. (39)	40	80%	68	NA	15/31	NA	NA	NA	NA	NA	no
Uboha, NV., et al. (25)	22	91%	64	5/19	NA	15/19	NA	NA	19/22	NA	yes
Qi, W.X., et al. (41)	20	95%	61.2	9/14	NA	NA	3/20	NA	14/20	NA	no
van Den Ende, T., et al. (13)	40	88%	63	10/33	NA	33/33	17/40	34/40	33/40	0	yes
Xu, X., et al. (28)	20	NA	NA	7/13	10/13	NA	7/13	NA	13/10	NA	no
Duan, H., et al. (29)	23	91%	63.5	6/17	9/17	16/17	7/23	21/23	17/23	0	yes
Gao, L., et al. (20)	20	85%	58.3	2/12	5/12	12/12	NA	20/20	12/20	0	yes
Gu, Y., et al. (30)	17	76%	65	4/15	8/15	15/15	6/17	17/17	15/17	NA	no
Guo, J., et al. (21)	15	NA	NA	NA	6/11	11/11	NA	NA	11/15	0	yes
He, W., et al. (31)	20	75%	61.4	3/16	7/16	14/16	4/20	18/20	16/20	0	yes
Jiang, B., et al. (22)	10	NA	NA	4/10	6/10	10/10	NA	NA	10/10	0	yes
Li, K., et al. (34)	17	94%	64	2/12	7/12	12/12	2/17	NA	12/17	0	yes
Li, Z., et al. (24)	20	80%	NA	3/20	7/20	20/20	2/20	NA	20/20	NA	yes
Liu, D., et al. (35)	23	NA	NA	6/18	NA	18/18	2/23	NA	18/23	NA	yes
Liu, J.; Li, J., et al. (36)	56	75%	61	16/51	30/51	51/51	6/56	51/56	51/56	NA	yes
Liu, J.; Yang, Y., et al. (37)	60	83%	65	20/51	35/51	50/51	34/60	55/60	51/60	8/60	yes
Ma, J., et al. (38)	42	NA	63	6/16	NA	NA	NA	NA	16/42	NA	no
Shang, X.,et al. (42)	49	NA	NA	12/29	21/29	29/29	0/29	NA	29/42	0	no
Shen, D., et al. (43)	28	96%	62.2	9/28	NA	26/27	2/28	28/28	27/28	0	yes
Wang, F., et al. (55)	26	65%	63	3/12	5/12	12/12	1/26	17/26	12/17	NA	no
Wang, W., et al. (26)	27	NA	NA	4/14	NA	14/14	NA	24/27	14/27	NA	no
Wang, Z., et al. (44)	30	80%	62	7/29	15/29	NA	11/30	29/30	29/30	5/30	yes
Xing, W., et al. (45)	30	73%	experiment group:63.8; control group:63.13	5/24	NA	24/24	NA	NA	24/30	NA	yes
Xu, W., et al. (56)	46	NA	NA	8/37	18/37	37/37	7/46	45/46	37/46	NA	not mentioned
Yamamoto, S.; Matsuda, S., et al. (40, 46)	25	NA	cohort A&B:62; cohort C&D:60	6/25	NA	23/25	NA	25/25	25/25	0	yes
Yan, X., et al. (47)	45	NA	NA	18/36	26/36	NA	15/45	NA	36/45	NA	yes
Yang, P., et al. (48)	16	88%	60.5	5/16	NA	15/16	NA	NA	16/16	NA	yes
Yang, W., et al. (49)	23	96%	58.6	5/20	10/20	20/20	NA	23/23	20/23	0	yes
Zhang, G., et al. (50)	24	NA	NA	3/18	9/18	NA	NA	NA	18/24	NA	no
Zhang, X (51).	25	NA	NA	8/25	16/25	NA	2/25	NA	25/25	0	yes
Zhang, Y., et al. (52)	166	84%	62.9	15/81	51/81	9/82	13/166	NA	82/166	NA	no
Zhang, Z.; Hong, Z., et al. (53)	30	87%	58.3	4/23	12/23	23/23	1/30	30/30	23/30	0	yes
Zhang, Z.; Ye, J., et al. (54)	40	NA	NA	10/40	19/40	39/40	NA	NA	NA	0	yes

pCR, pathological complete response; MPR, major pathological response; TRAEs, treatment-related adverse events. NA, not available.

### 3.2 Quality assessment

**Figure 2:**
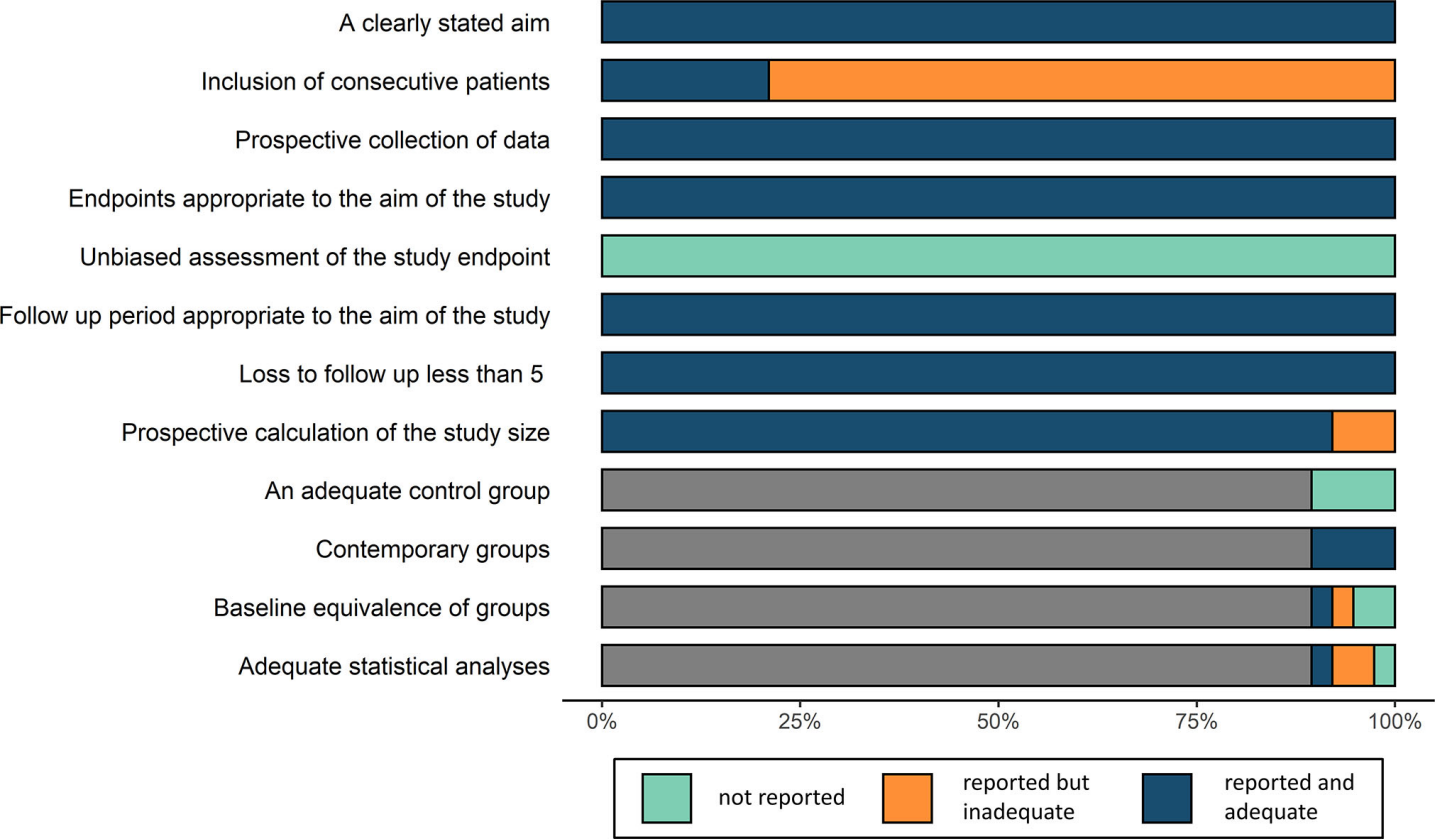
Quality assessment by the methodological index for non-randomized studies (MINORS) index.

The 38 studies were scored from 13 to 18 using the MINORS index, with a low risk of summary bias, indicating acceptable quality for the present meta-analysis (**Figure 2**; **Supplementary Table 1**).

### 3.3 Efficacy

#### 3.3.1 Pathological complete responses

**Figure 3:**
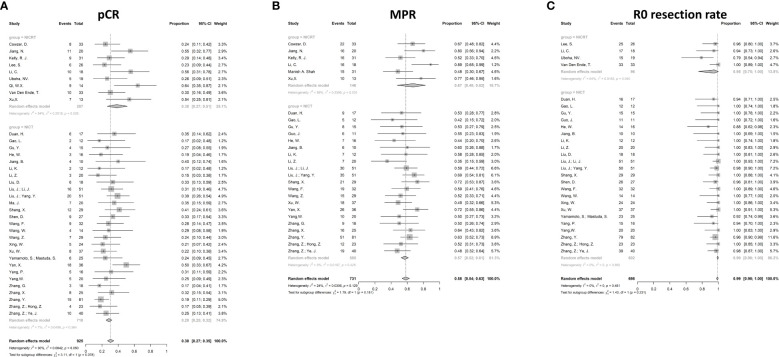
Forest plots for efficacy. **(A)** Pathological complete response (pCR), **(B)** Major pathological response (MPR), and **(C)** R0 resection rate. NICRT, neoadjuvant immune-chemoradiotherapy; NICT, neoadjuvant immune-chemotherapy.

The pooled pCR for 925 patients in 36 studies with available pCR data was 30% (95%CI 27%-35%) with potential heterogeneity (I^2^ = 30%, p=0.050) (**Figure 3A**). The pCR in the NICRT group and NICT group was 38% (95%CI 27%-51%) and 28% (95%CI 25-32%), respectively. Although numerically higher in the NICRT group, no significant difference was shown between the two groups (p=0.078). In addition, high heterogeneity was observed in the NICRT group (I^2^ = 60%, p=0.026) however not in the NICT group (I^2^ = 11%, p=0.364).

#### 3.3.2 Major pathological responses

The MPR data were available in 27 studies. The pooled MPR was 58% (95%CI 54%-63%, I^2^ = 24%, p=0.129) (**Figure 3B**). The pooled MPR in the NICRT group was 67% (95%CI 48%-82%, I^2^ = 59%, p=0.031)., while the pooled MPR in the NICT group was 57% (95%CI 52%-61%, I^2^ = 3%, p=0.425). There were no statistical differences between the two groups (p=0.181).

#### 3.3.3 R0 resection rate

Pooled analysis showed a high R0 resection rate reaching 99% in 27 studies (95%CI 98%-100%, I^2^ = 0%, p=0.862) (**Figure 3C**). Both the NICRT and NICT groups achieved a high chance of R0 resection (95% and 99%, respectively).

### 3.4 Safety

#### 3.4.1 Incidence of ≥grade 3 TRAEs

**Figure 4:**
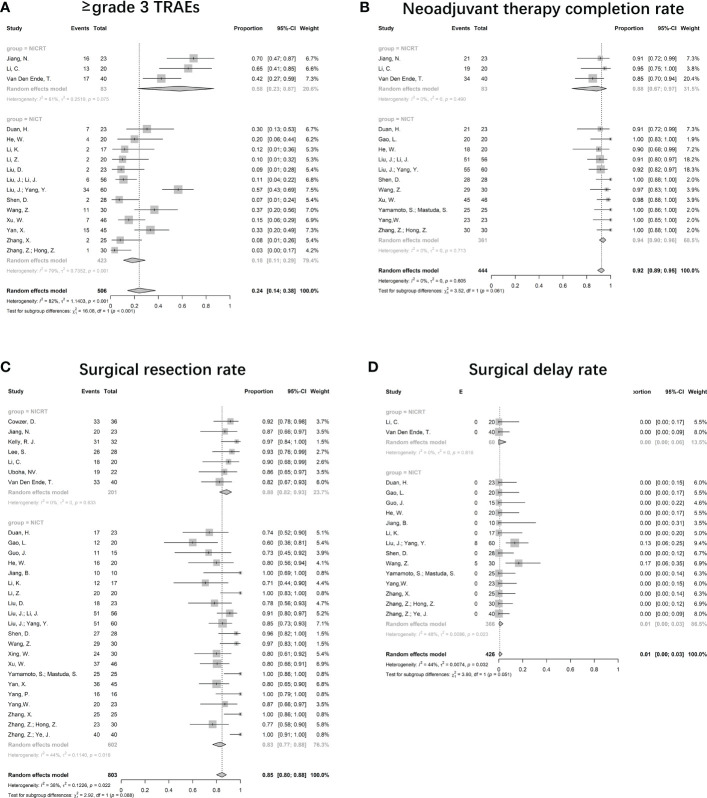
Forest plots for safety. **(A)** Incidence of ≥grade 3 TRAEs, **(B)** Neoadjuvant therapy completion rate, **(C)** Surgical resection rate, and **(D)** Surgical delay rate. NICRT, neoadjuvant immune-chemoradiotherapy; NICT, neoadjuvant immune-chemotherapy.

As the overall incidence of TRAEs has not been widely reported, we analyzed ≥grade 3 TRAEs. The pooled incidence was 24% (95%CI 14%-38%) (**Figure 4A**). 16 studies with 506 patients provided valid data. Significant heterogeneity was observed (I^2^ = 82%, p<0.001). Most of the reported ≥grade 3 TRAEs were hematologic, including lymphopenia, leukopenia, thrombocytopenia, and anemia. Non-hematological TRAEs, such as anorexia, rash, vomiting, and diarrhea, were less common. Grade 5 TRAEs were rare and were mainly due to hemorrhage. Although significant differences were shown between the NICRT and NICT groups (58%, 95%CI 23%-87% vs. 18%, 95%CI 11%-29%, p<0.001), high heterogeneity was found in both groups (I^2^ = 61%, p=0.075 vs. I^2^ = 79%, p<0.001).

#### 3.4.2 Neoadjuvant therapy completion rates

Valid data were reported in 14 studies. The NTCR had a pooled value of 92% (95%CI 89%-95%, I^2^ = 0%; p=0.605) (**Figure 4B**). The pooled NTCR was similarly high in both the NICRT and NICT groups (88% vs. 94%, p=0.061).

#### 3.4.3 Surgical resection rate

28 trials provided valid data, leading to a pooled rate of 85% (95%CI 80%-88%, I^2^ = 38%, p=0.022) (**Figure 4C**). The surgical resection rates in the NICRT vs NICT groups were 88% (95%CI 82%-93%, I^2^ = 0%, p=0.633) vs 83% (95%CI 77%-88%, I^2^ = 44%, p=0.018), with no significant differences (p=0.088).

#### 3.4.4 Surgical delay rate

Among the 16 valid studies, only 2 reported a surgical delay rate of 13% and 17%, and the others reported no surgical delay. The pooled surgical delay rate was 1% (95%CI 0%-3%, I^2^ = 44%, p=0.032) (**Figure 4D**).

### 3.5 Efficacy and safety in ESCC and EAC

**Figure 5:**
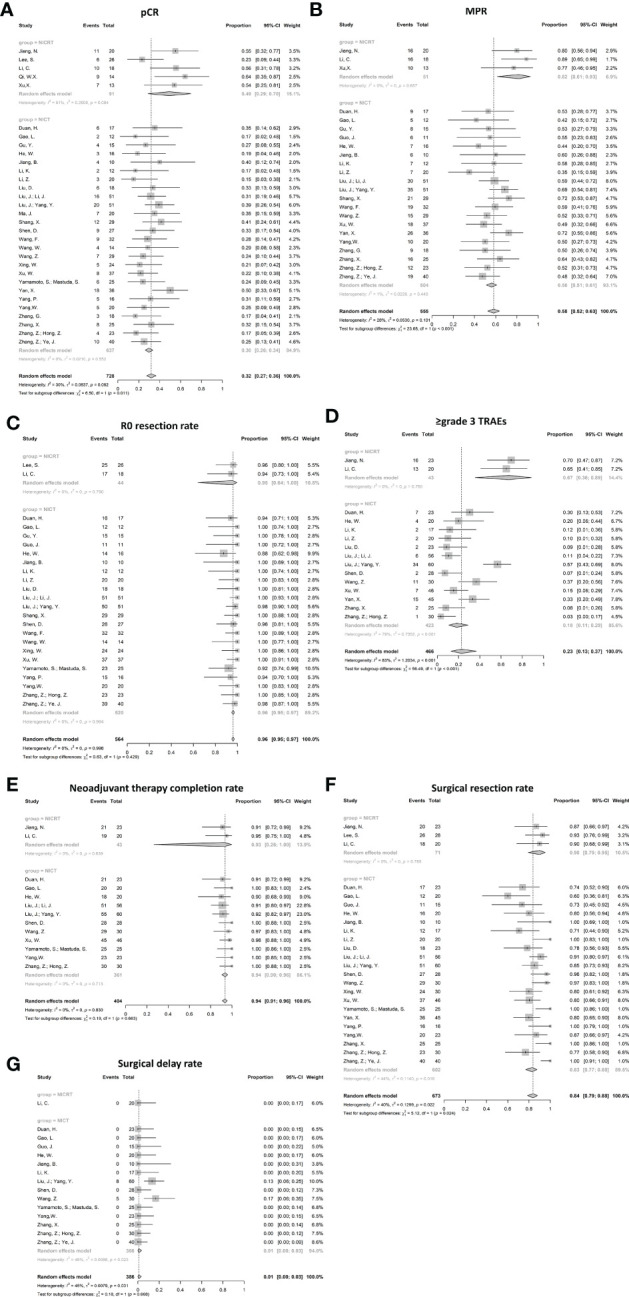
Forest plots of efficacy and safety in ESCC. **(A)** Pathological complete response (pCR), **(B)** Major pathological response (MPR), **(C)** R0 resection rate, **(D)** Incidence of ≥grade 3 TRAEs, **(E)** Neoadjuvant therapy completion rate, **(F)** surgical resection rate, and **(G)** Surgical delay rate. NICRT, neoadjuvant immune-chemoradiotherapy; NICT, neoadjuvant immune-chemotherapy.

32 (84%) of the included studies enrolled patients only with ESCC. A pooled analysis was performed, specifically for ESCC patients (**Figure 5**). 5 studies were in the NICRT group and 27 were in the NICT group. The overall pooled pCR was 32% (95%CI 27%-36%, I^2^ = 30%, p=0.062). The pooled pCR in the NICRT and NICT groups was 49% (95%CI 29%-70%, I^2^ = 51%, p=0.084) and 30% (95%CI 26%-34%, I^2^ = 0%, p=0.552), respectively, with significant differences (p=0.011). The pooled MPR was 58% (95%CI 52%-63%, I^2^ = 28%, p=0.101). The pooled MPR in the NICRT and NICT groups was 82% (95%CI 61%-93%, I^2^ = 0%, p=0.657) and 56% (95%CI 51%-61%, I^2^ = 1%, p=0.440), respectively, with significant differences (p<0.001). The R0 resection rate was 96% (95%CI 95%-97%, I^2^ = 0%, p=0.998), and no significant differences were shown between the NICRT and NICT groups (95% vs. 96%, p=0.429). The pooled incidence of ≥ grade 3 TRAEs was 23% (95%CI 13%-37%, I^2^ = 83%, p<0.001). Incidence of ≥ grade 3 TRAEs in the NICRT and NICT group was 67% (95%CI 36%-89%, I^2^ = 0%, p=0.750) and 18% (95%CI 11%-29%, I^2^ = 79%, p<0.001), respectively, with a significant difference (p<0.001). The pooled NTCR was 94% (95%CI 91%-96%, I^2^ = 0%, p=0.830), with 93% for the NICRT group and 94% for NICT group. The surgical resection rate was 84% (95%CI 79%-88%, I^2^ = 40%, p=0.022). The NICRT group had a higher surgical resection rate than did the NICT group (90% vs. 83%, p=0.024). The surgical delay rate was 1% (95%CI 0%-3%, I^2^ = 45%, p=0.031).

**Figure 6:**
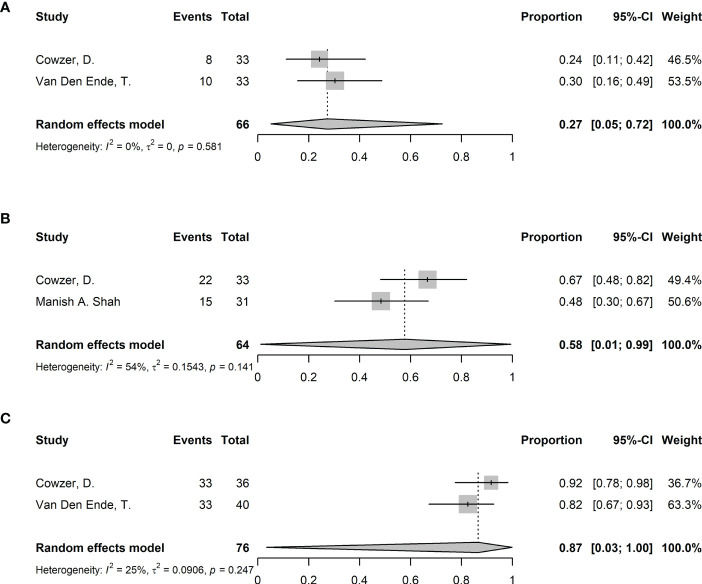
Forest plots of efficacy and safety in EAC. **(A)** Pathological complete response (pCR), **(B)** Major pathological response (MPR), and **(C)** surgical resection rate.

3 (8%) of the included studies enrolled patients only with EAC. All 3 studies adopted NICRT. The pooled analysis was only performed for outcomes with more than 1 available study (i.e., pCR, MPR and surgical resection rate) (**Figure 6**). The pooled pCR was 27% (95%CI 5%-72%, I^2^ = 0%, p=0.581). The pooled MPR was 58% (95%CI 1%-99%, I^2^ = 54%, p=0.141). The pooled surgical resection rate was 87% (95%CI 3%-100%, I^2^ = 25%, p=0.247). The R0 resection rate, incidence of ≥ grade 3 TRAEs, NTCR and surgical delay rate were reported in single study as 100%, 42.5%, 85%, 0%.

### 3.6 Exploratory subgroup analysis

Subgroup analysis was performed based on types of publication (abstract or article), types of ICI and cycles of ICI. The cycles of ICI were categorized into 2 subgroups, i.e., 2 cycles and >2 cycles (including studied adopting 2 or more cycles, such as 2-4 cycles). Studies not reporting relevant factors were excluded. Due to the level of heterogeneity and incidence of outcomes, only pCR, MPR, incidence of ≥ grade 3 TRAEs and surgical resection rate underwent subgroup analysis. Findings indicate the types of publication and the cycles of ICI were not source of heterogeneity (**eFigure 1** and **eFigure 2** in Supplement). The types of ICI might affect MPR (p=0.017), with relatively high MPR for tislelizumab (72%, 95%CI 55%-86%) and low MPR for sintilimab (49%, 95%CI 41%-56%) (**eFigure 3B** in Supplement). The types of ICI might also contribute to the heterogeneity of incidence of ≥ grade 3 TRAEs (p=0.023), with relatively high incidence for pembrolizumab (65%, 95%CI 41%-85%) and atezolizumab (42%, 95%CI 27-59%) and low incidence for sintilimab (13%, 95%CI 1-77%) (**eFigure 3C** in Supplement). Surgical resection rate might also be affected by types of ICI (p<0.001), with relatively high rate for nivolumab (97%, 95%CI 65%-100%) and low rate for toripalimab (76%, 95%CI 65%-84%) (**eFigure 3D** in Supplement).

### 3.7 Survival

The survival data were mostly incomplete or immature. Only a few trials published limited data, which was insufficient for the pooled analysis. According to the reported data (**Table 3**), one study reported a median OS of 29.7 months (13), and the median DFS ranged from 19.4 to 35.4 months (13, 32). The 1-year OS rate ranged from 77% to 92% (19, 25, 33, 48), and the 2-year OS rate ranged from 73.1% to 85% (19, 33).The 1-year DFS rate ranged from 67% to 83% (19, 25, 48). Onestudy reported a 2-year DFS rate of 71% (19).

**Table 3:** Survival data.

Study	Median follow-up, months	Median OS, months (95%CI)	Median DFS, months (95%CI)	1y OS (95%CI)	1y DFS (95%CI)	2y OS (95%CI)	2y DFS (95%CI)
Cowzer, D., et al. (19)	30	NA	NA	92% (83%-100%)	81% (69%-95%)	85% (74%-98%)	71% (58%-88%)
Kelly, R. J., et al (32),	30	NA	35.4 (24.7-NA)	NA	79.1% (65.5-95.6%)	NA	NA
Lee, S., et al. (33)	NA	NA	NA	80.80%	NA	73.10%	NA
Uboha, NV., et al. (25)	9.8	NA	NA	77%	67%	NA	NA
van den Ende, T., et al. (13)	24	29.7	19.4	NA	NA	NA	NA
Yang, P., et al. (48)	NA	NA	NA	90.90%	83%	NA	NA

OS, overall survival; DFS, disease-free survival; NA, not available.

### 3.8 Sensitivity analysis, publication bias and certainty assessment

**Figure 7:**
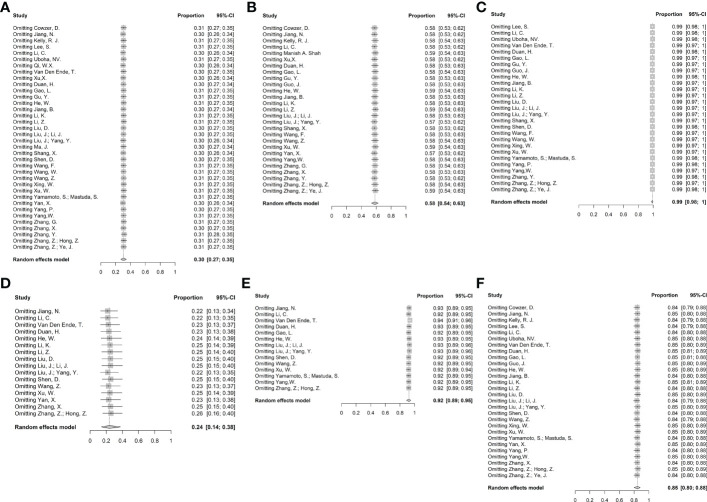
Sensitivity analysis. **(A)** pCR, **(B)** MPR, **(C)** R0 resection rate, **(D)** Incidence of ≥ grade 3 TRAEs, **(E)** Neoadjuvant therapy completion rate, and **(F)** Surgical resection rate.

Sensitivity analysis revealed no obvious changes regarding efficacy and safety when excluding any single study (**Figure 7**). Results of Egger’s and Begg’s tests were shown in **Supplementary Table 2**. No publication bias was detected in the pCR or MPR groups. The incidence of ≥3 TRAEs, NTCR, and surgical resection rates exhibited a publication bias in both tests. Furthermore, there was a discrepancy between the Begg’s test and Egger’s test in terms of the surgical delay rate (p=0.005 vs. p=0.093) and R0 resection rate (p=0.030 vs. p=0.372). Considering that Egger’s test is more sensitive (57), potential publication bias might not exist in these outcomes. The details of certainty assessment are shown in **eTable 1** in Supplement. Notably, due to no RCTs were included, the certainty was graded from low certainty, and downgraded if certain limitations were met. Overall, the results of pCR, MPR and R0 resection rate had low certainty of evidence, and the results of incidence of ≥ grade 3 TRAEs, NTCR and surgical resection rate had very low certainty of evidence.

## 4 Discussion

Overall, these results support the acceptable efficacy and safety of neoadjuvant immunotherapy combined with chemoradiotherapy or chemotherapy for esophageal cancer. To our knowledge, this is the largest meta-analysis of neoadjuvant immunotherapy for esophageal cancer based on prospective studies.

The pooled pCR reached 30%, a value much higher than the pCR of 0%-9% of neoadjuvant chemotherapy mentioned in previous RCTs (58, 59), and even higher than the latest DCF neoadjuvant chemotherapy regimen discussed in JCOG1109, mentioning a pCR of 19.8% (60). Moreover, the pooled pCR was comparable to the 29% in the neoadjuvant chemoradiotherapy group in the CROSS trial (3). A high pooled MPR (58%) also demonstrated good efficacy. The pooled R0 resection rate reached 99%, which is close to the 92%-98.4% for neoadjuvant chemoradiotherapy in the CROSS and NEOCRTEC5010 trials (3, 4), and higher than the reported 74%-89% for neoadjuvant chemotherapy (8), indicating a satisfying guarantee for high-quality surgery. These results showed that the short-term efficacy of neoadjuvant immunotherapy was favorable.

It remains unclear how pCR or MPR affect the survival of patients with esophageal cancer receiving neoadjuvant immunotherapy. Due to insufficient follow-up, survival data were not available for most trials. Based on the limited data, the 1-year and 2-year OS for neoadjuvant immunotherapy were comparable to the 1-year and 2-year OS being 81-90% and 67-75.1% for neoadjuvant chemoradiotherapy in different RCTs (4, 5). DFS rates were similar to those of neoadjuvant chemoradiotherapy. In the PERFECT trial, no significant difference in OS or DFS was observed between the neoadjuvant immunotherapy cohort and the propensity score-matched neoadjuvant chemoradiotherapy cohort (13). Briefly, neoadjuvant immunotherapy may achieve a short-term survival similar to that achieved with neoadjuvant chemoradiotherapy. However, large-scale RCTs with long follow-up periods are necessary to further investigate survival, particularly long-term survival.

The adverse event profiles were acceptable. The pooled overall incidence of ≥grade 3 TRAEs was 24%. In comparison, the incidence of ≥ grade 3 leukopenia in the neoadjuvant chemotherapy group in Intergroup0113 trial was 29% (59), and the incidence of ≥ grade 3 neutropenia related to preoperative chemotherapy was 23.8% in the MAGIC trial (61), indicating that the addition of immunotherapy might not increase the incidence of serious adverse events. Regarding neoadjuvant chemoradiotherapy, the incidence of ≥ grade 3 TRAEs was 17%-27.6% in the FFCD9901 and CROSS trials (3, 62), and 54.3% of patients developed ≥ grade 3 hematologic toxicity in the NEOCRTEC 5010 trial (4), supporting the fact that the incidence of severe adverse events for neoadjuvant immunotherapy may not be significantly higher than that of chemoradiotherapy. Meanwhile, the incidence of ≥ grade 3 immune-related adverse events was as low as 2.5%-17.8% in the included studies, which supported the safety of immunotherapy. In addition, almost all patients (92%) completed the entire course of neoadjuvant immunotherapy, indicating that the addition of ICIs may not affect the overall administration of the full regimen, and adverse events may be clinically rectifiable to continue the treatment course. The pooled surgical resection rate was close to 82%-93% reported in studies on neoadjuvant chemoradiotherapy (3, 4, 62), and surgery delay was rare. Neoadjuvant immunotherapy combined with chemoradiotherapy or chemotherapy is generally safe. Nevertheless, publication bias and heterogeneity made the results unstable, so caution should be exercised when drawing conclusions. Large clinical trials are required to validate its safety.

The pCR and MPR of the NICRT group were both higher than those of the NICT group numerically. Although a significant difference between the two groups was not observed, it might have been underestimated. First, the efficacy of neoadjuvant treatment may be better in ESCC than in EAC (3), and most patients in the NICT group had ESCC, whereas some patients in the NICRT group had EAC. This can also explain why the pCR and MPR of the NICRT group showed apparent heterogeneity among the included studies. Second, the p-value may have been affected by the number of patients in the NICRT group. Notably, the incidence of ≥ grade 3 TRAEs was significantly higher in the NICRT group than in the NICT group, indicating that the intense treatment modality might increase the risk of adverse events. However, high heterogeneity existed, which may have been caused by differences in the treatment regimen and follow-up time.

ESCC had a higher pCR than EAC in the CROSS trial (3). The KEYNOTE-181 and CHECKMATE-577 trials showed that ESCC responded better to immunotherapy than EAC (11, 63). In addition, ESCC is more radiosensitive (64); therefore, it may benefit more from neoadjuvant immunotherapy, especially in the NICRT group. Our specifical subgroup analysis of ESCC showed that neoadjuvant immunotherapy was also effective. In the ESCC subgroup, the pooled pCR and MPR were 49% and 82%, respectively, which were significantly higher than NICT, and close to the pCR of 43.2%-49% for ESCC patients receiving neoadjuvant chemoradiotherapy (3, 4). Notably, except for the pCR of 23% reported by Lee et al. (33), all studies in the NICRT group had a pCR above 54%. Whether NICRT can lead to better short-term efficacy than neoadjuvant chemoradiotherapy requires further investigation in RCTs. Patients with EAC undergoing NICRT had pooled pCR of 27%, comparing to 23% for EAC in the CROSS trial (3), with satisfactory MPR and surgical resection rate. However, the available studies were too few, and more research must be conducted to explore the value of neoadjuvant immunotherapy for EAC.

Exploratory subgroup analysis indicated that the types of ICI might be associated with the efficacy and safety of neoadjuvant immunotherapy for esophageal cancer, which may cause heterogeneity of outcomes. However, due to the limited sample size in each drug group, more relevant clinical trials are necessary to explore the correlation further.

Neoadjuvant immunotherapy combined with radiotherapy is not a common choice for esophageal cancer, as only one trial has published the results in an abstract. Of the 14 patients who underwent surgery, seven (50%) achieved pCR and nine (64.3%) achieved MPR. Only 1 patient developed ≥ grade 3 TRAEs among the 22 patients, although some patients were still waiting for surgery (65). This modality can possibly achieve favorable efficacy with a low incidence of adverse events. Notably, it has been demonstrated that neoadjuvant immunotherapy combined with radiotherapy achieved higher pCR (26.7%) and MPR (53.3%) than immunotherapy alone, with similar toxicity (66). This modality is promising, but the exploration and selection of suitable specific patient groups based on biomarkers (e.g., PD-L1 expression) is required.

This meta-analysis had several limitations. Although the number of trials was adequate, most studies were early phase trials with a relatively small sample size. The risk of selection bias existed due to the selected cohorts adopted in the trials, which constrained reliability. The results of outcomes can only provide low or very low certainty of evidence. Some trials are still ongoing; therefore, the final outcome may not be consistent with the interim readout. The majority of trials were published in abstracts, making it difficult to access complete data. Furthermore, because of the relatively small number of studies and patients in the NICRT group, the statistical differences between NICRT and NICT should be interpreted with caution. Most trials have focused on ESCC; therefore, the interpretation was not reliable with regard to EAC. Although a specifical analysis were performed for EAC, the results were not able to lead to any meaningful deduction due to too few data. In addition, variations exist among different trials in the treatment regimen, study design, and patients’ characteristics. However, most outcomes showed acceptable heterogeneity and produced relatively stable results in sensitivity analysis, supporting the reliability of results. Finally, another limitation is the lack of long-term survival data, which has not yet been reported in most trials. Due to the small sample size and short follow-up time, it is hard to obtain reliable survival results based on current data.

This meta-analysis illustrates that neoadjuvant immunotherapy combined with chemoradiotherapy or chemotherapy can be effective and safe in the short term for locally advanced esophageal cancer, especially for ESCC, and can be used as a reference for future trials. The value of neoadjuvant immunotherapy for EAC needs more research to explore. NICRT may achieve better short-term efficacy with possibly higher risk of adverse events than NICT for ESCC. However, whether NICRT or NICT is more favorable for locally advanced esophageal cancer merits further investigation.

## Data availability statement

The original contributions presented in the study are included in the article/**Supplementary Material**. Further inquiries can be directed to the corresponding author.

## Author contributions

ZH and YL conceived the study. YL and YB performed the searches and collected raw data. XY, SS, MY, ZM and YW checked the data and performed statistical analysis. WZ and YZ made substantial contributions to interpretation of data. YL, WZ, YB, XY, SS, MY, ZM and YW drafted the manuscript. YZ, YM, JQ, LX, JW and ZH provided important expertise on design and revised the whole manuscript critically. All authors contributed to the article and approved the submitted version.

## References

[1] Bray F Ferlay J Soerjomataram I Siegel RL Torre LA Jemal A . Global cancer statistics 2018: GLOBOCAN estimates of incidence and mortality worldwide for 36 cancers in 185 countries. CA Cancer J Clin (2018) 68(6):394–424. doi: 10.3322/caac.21492 30207593

[2] Siegel RL Miller KD Jemal A . Cancer statistics, 2020. CA Cancer J Clin (2020) 70(1):7–30. doi: 10.3322/caac.21590 31912902

[3] van Hagen P Hulshof MC van Lanschot JJ Steyerberg EW van Berge Henegouwen MI Wijnhoven BP . Preoperative chemoradiotherapy for esophageal or junctional cancer. N Engl J Med (2012) 366(22):2074–84. doi: 10.1056/NEJMoa1112088 22646630

[4] Yang H Liu H Chen Y Zhu C Fang W Yu Z . Neoadjuvant chemoradiotherapy followed by surgery versus surgery alone for locally advanced squamous cell carcinoma of the esophagus (NEOCRTEC5010): A phase III multicenter, randomized, open-label clinical trial. J Clin Oncol (2018) 36(27):2796–803. doi: 10.1200/JCO.2018.79.1483 PMC6145832 30089078

[5] Shapiro J van Lanschot JJB Hulshof MCCM van Hagen P van Berge Henegouwen MI Wijnhoven BPL . Neoadjuvant chemoradiotherapy plus surgery versus surgery alone for oesophageal or junctional cancer (CROSS): long-term results of a randomised controlled trial. Lancet Oncol (2015) 16(9):1090–8. doi: 10.1016/S1470-2045(15)00040-6 26254683

[6] Ando N Kato H Igaki H Shinoda M Ozawa S Shimizu H . A randomized trial comparing postoperative adjuvant chemotherapy with cisplatin and 5-fluorouracil versus preoperative chemotherapy for localized advanced squamous cell carcinoma of the thoracic esophagus (JCOG9907). Ann Surg Oncol (2012) 19(1):68–74. doi: 10.1245/s10434-011-2049-9 21879261

[7] Al-Batran SE Homann N Pauligk C Goetze TO Meiler J Kasper S . Perioperative chemotherapy with fluorouracil plus leucovorin, oxaliplatin, and docetaxel versus fluorouracil or capecitabine plus cisplatin and epirubicin for locally advanced, resectable gastric or gastro-oesophageal junction adenocarcinoma (FLOT4): a randomised, phase 2/3 trial. Lancet (2019) 393(10184):1948–57. doi: 10.1016/S0140-6736(18)32557-1 30982686

[8] Jing SW Qin JJ Liu Q Zhai C Wu YJ Cheng YJ . Comparison of neoadjuvant chemoradiotherapy and neoadjuvant chemotherapy for esophageal cancer: a meta-analysis. Future Oncol (2019) 15(20):2413–22. doi: 10.2217/fon-2019-0024 31269806

[9] Bang YJ Kang YK Catenacci DV Muro K Fuchs CS Geva R . Pembrolizumab alone or in combination with chemotherapy as first-line therapy for patients with advanced gastric or gastroesophageal junction adenocarcinoma: results from the phase II nonrandomized KEYNOTE-059 study. Gastric Cancer (2019) 22(4):828–37. doi: 10.1007/s10120-018-00909-5 PMC6570680 30911859

[10] Shah MA Kojima T Hochhauser D Enzinger P Raimbourg J Hollebecque A . Efficacy and safety of pembrolizumab for heavily pretreated patients with advanced, metastatic adenocarcinoma or squamous cell carcinoma of the esophagus: The phase 2 KEYNOTE-180 study. JAMA Oncol (2019) 5(4):546–50. doi: 10.1001/jamaoncol.2018.5441 PMC6459121 30570649

[11] Kojima T Shah MA Muro K Francois E Adenis A Hsu CH . Randomized phase III KEYNOTE-181 study of pembrolizumab versus chemotherapy in advanced esophageal cancer. J Clin Oncol (2020) 38(35):4138–48. doi: 10.1200/JCO.20.01888 33026938

[12] Li C Zhao S Zheng Y Han Y Chen X Cheng Z . Preoperative pembrolizumab combined with chemoradiotherapy for oesophageal squamous cell carcinoma (PALACE-1). Eur J Cancer (Oxford England: 1990) (2021) 144:232–41. doi: 10.1016/j.ejca.2020.11.039 33373868

[13] van den Ende T de Clercq NC van Berge Henegouwen MI Gisbertz SS Geijsen ED Verhoeven RHA . Neoadjuvant chemoradiotherapy combined with atezolizumab for resectable esophageal adenocarcinoma: A single-arm phase II feasibility trial (PERFECT). Clin Cancer Res (2021) 27(12):3351–9. doi: 10.1158/1078-0432.CCR-20-4443 33504550

[14] Hutton B Salanti G Caldwell DM Chaimani A Schmid CH Cameron C . The PRISMA extension statement for reporting of systematic reviews incorporating network meta-analyses of health care interventions: checklist and explanations. Ann Intern Med (2015) 162(11):777–84. doi: 10.7326/M14-2385 26030634

[15] Hellmann MD Chaft JE William WN Rusch V Pisters KMW Kalhor N . Pathological response after neoadjuvant chemotherapy in resectable non-small-cell lung cancers: proposal for the use of major pathological response as a surrogate endpoint. Lancet Oncol (2014) 15(1):e42–50. doi: 10.1016/S1470-2045(13)70334-6 PMC4734624 24384493

[16] Pataer A Kalhor N Correa AM Raso MG Erasmus JJ Kim ES . Histopathologic response criteria predict survival of patients with resected lung cancer after neoadjuvant chemotherapy. J Thorac Oncol (2012) 7(5):825–32. doi: 10.1097/JTO.0b013e318247504a PMC3465940 22481232

[17] Slim K Nini E Forestier D Kwiatkowski F Panis Y Chipponi J . Methodological index for non-randomized studies (minors): development and validation of a new instrument. ANZ J Surg (2003) 73(9):712–6. doi: 10.1046/j.1445-2197.2003.02748.x 12956787

[18] Guyatt GH Oxman AD Schünemann HJ Tugwell P Knottnerus A . GRADE guidelines: a new series of articles in the journal of clinical epidemiology. J Clin Epidemiol (2011) 64(4):380–2. doi: 10.1016/j.jclinepi.2010.09.011 21185693

[19] Cowzer D Wu AJ-C Sihag S Walch HS Park BJ Jones DR . Durvalumab (D) and PET-directed chemoradiation (CRT) after induction FOLFOX for esophageal adenocarcinoma: Final results. J Clin Oncol (2022) 40(16_suppl):4029. doi: 10.1200/JCO.2022.40.16_suppl.4029

[20] Gao L Lu J Zhang P Hong ZN Kang M . Toripalimab combined with docetaxel and cisplatin neoadjuvant therapy for locally advanced esophageal squamous cell carcinoma: a single-center, single-arm clinical trial (ESONICT-2). J Gastrointest Oncol (2022) 13(2):478–87. doi: 10.21037/jgo-22-131 PMC9086050 35557591

[21] Guo J ed. Neoadjuvant sintilimab combined with chemotherapy in patients with resectable esophageal squamous cell carcinoma (ESCC): Preliminary results from a phase II study2022, in: ASCO Annual Meeting, JCO: American Society of Clinical Oncology. (2022) 40(16_suppl):e16008.

[22] Jiang B Yang X Zhang J Huang M . Abstract 5230: Neoadjuvant programmed cell death protein 1 inhibitors combined with chemotherapy in resectable esophageal squamous carcinoma: an open-label, single-arm study. Cancer Res (2022) 82(12_Supplement):5230. doi: 10.1158/1538-7445.AM2022-5230

[23] Jiang N ed. SCALE-1: Safety and efficacy of short course neoadjuvant chemo-radiotherapy plus toripalimab for locally advanced resectable squamous cell carcinoma of esophagus2022, in: ASCO Annual Meeting, JCO: American Society of Clinical Oncology (2022) 40(16_suppl):4063.

[24] Li Z ed. A study of neoadjuvant sintilimab combined with chemotherapy TP for locally advanced esophageal squamous cell carcinoma (ESCC)2022, in: ASCO Annual Meeting, JCO: American Society of Clinical Oncology (2022) 40(16_suppl):e16038.

[25] Uboha NV Eickhoff JC Maloney JD McCarthy D DeCamp M Deming DA . Phase I/II trial of perioperative avelumab in combination with chemoradiation (CRT) in the treatment of stage II/III resectable esophageal and gastroesophageal junction (E/GEJ) cancer. J Clin Oncol (2022) 40(16_suppl):4034. doi: 10.1200/JCO.2022.40.16_suppl.4034 PMC12186108 39757733

[26] Wang W ed. Neoadjuvant pembrolizumab plus chemotherapy for resectable locally advanced esophageal squamous cell carcinoma (ESCC): Interim results2022, in: ASCO Annual Meeting, JCO: American Society of Clinical Oncology (2022) 40(16_suppl):e16011.

[27] Xu L Qi Y Jiang Y Ji Y Zhao Q Wu J . Crosstalk between the gut microbiome and clinical response in locally advanced thoracic esophageal squamous cell carcinoma during neoadjuvant camrelizumab and chemotherapy. Ann Trans Med (2022) 10(6):325. doi: 10.21037/atm-22-1165 PMC9011252 35433940

[28] Xu X ed. Neoadjuvant chemoradiotherapy combined with perioperative toripalimab in locally advanced esophageal cancer2022, in: ASCO Annual Meeting, JCO: American Society of Clinical Oncology (2022) 40(16_suppl):e16065.

[29] Duan H Wang T Luo Z Wang X Liu H Tong L . A multicenter single-arm trial of sintilimab in combination with chemotherapy for neoadjuvant treatment of resectable esophageal cancer (SIN-ICE study). Ann Trans Med (2021) 9(22):1700. doi: 10.21037/atm-21-6102 PMC8667140 34988209

[30] Gu Y Chen X Wang D Ding M Xue L Zhen F . A study of neoadjuvant sintilimab combined with triplet chemotherapy of lipo-paclitaxel, cisplatin, and s-1 for resectable esophageal squamous cell carcinoma (ESCC). Ann Oncol (2020) 31:S1307–S8. doi: 10.1016/j.annonc.2020.10.196

[31] He W Leng X Mao T Luo X Zhou L Yan J . Toripalimab plus paclitaxel and carboplatin as neoadjuvant therapy in locally advanced resectable esophageal squamous cell carcinoma. oncologist (2022) 27(1):e18–28. doi: 10.1093/oncolo/oyab011 PMC8842349 35305102

[32] Kelly RJ Zaidi AH van Liere Canzoniero J Feliciano JL Hales RK Voong KR . Multicenter phase II study of neoadjuvant nivolumab or nivolumab plus relatlimab (antiLAG3 antibody) plus chemoradiotherapy in stage II/III esophageal/gastroesophageal junction (E/GEJ) carcinoma. J Clin Oncol (2022) 40(4 SUPPL):321. doi: 10.1200/JCO.2022.40.4_suppl.321

[33] Lee S Ahn BC Park SY Kim DJ Lee CG Cho J . A phase II trial of preoperative chemoradiotherapy and pembrolizumab for locally advanced esophageal squamous cell carcinoma (ESCC). Ann Oncol (2019) 30:v754. doi: 10.1093/annonc/mdz065.004

[34] Li K Yang X Luo W Ma Q Wang Y Xiong Y . Toripalimab plus nab-paclitaxel and carboplatin as neoadjuvant therapy for patients with esophageal squamous cell carcinoma at clinical stage t2-t4/n0-n2/m0: A single-arm, single-center clinical study. J ImmunoTher Cancer (2020) 8(SUPPL 3):A253. doi: 10.1136/jitc-2020-SITC2020.0415

[35] Liu D Zhang Q Zhu J Qian T Yin R Fan Z . Phase-II study of toripalimab combined with neoadjuvant chemotherapy for the treatment of resectable esophageal squamous cell carcinoma. J Clin Oncol (2021) 39(15 SUPPL):e16029. doi: 10.1200/JCO.2021.39.15_suppl.e16029

[36] Liu J Li J Lin W Shao D Depypere L Zhang Z . Neoadjuvant camrelizumab plus chemotherapy for resectable, locally advanced esophageal squamous cell carcinoma (NIC-ESCC2019): A multicenter, phase 2 study. Int J Cancer (2022) 151(1):128–37. doi: 10.1002/ijc.33976 35188268

[37] Liu J Yang Y Liu Z Fu X Cai X Li H . Multicenter, single-arm, phase II trial of camrelizumab and chemotherapy as neoadjuvant treatment for locally advanced esophageal squamous cell carcinoma. J immunother Cancer (2022) 10(3):e004291. doi: 10.1136/jitc-2021-004291 35338088 PMC8961177

[38] Ma J Zhang J Yang Y Zheng D Wang X Liang H . 65P camrelizumab combined with paclitaxel and nedaplatin as neoadjuvant therapy for locally advanced esophageal squamous cell carcinoma (ESPRIT): A phase II, single-arm, exploratory research. Ann Oncol (2021) 32:S1400. doi: 10.1016/j.annonc.2021.10.083

[39] Manish A Shah KA . Multicenter, randomized phase II study of neoadjuvant pembrolizumab plus chemotherapy and chemoradiotherapy in esophageal adenocarcinoma (EAC)2021, in: ASCO Annual Meeting, JCO: American Society of Clinical Oncology (2021) 39(15_suppl):4005.

[40] Matsuda S Yamamoto S Kato K Daiko H Kojima T Hara H . FRONTiER: A feasibility trial of nivolumab with neoadjuvant CF or DCF, FLOT therapy for locally advanced esophageal carcinoma (JCOG1804E)-short-term results for cohorts c and d. J Clin Oncol (2022) 40(4 SUPPL):286. doi: 10.1200/JCO.2022.40.4_suppl.286

[41] Qi WX Zhao S Li H Chen J . Safety and tolerability of neoadjuvant chemoradiotherapy combined with pembrolizumab for local advanced, resectable esophageal cancer: preliminary results of a prospective phase IB trial. Int J Radiat Oncol Biol Phys (IJROBP) (2020) 108(3):e576–e7. doi: 10.1016/j.ijrobp.2020.07.1773

[42] Shang X Zhang C Zhao G Zhang W Liu L Duan X . LBA3 safety and efficacy of pembrolizumab combined with paclitaxel and cisplatin as a neoadjuvant treatment for locally advanced resectable (stage III) esophageal squamous cell carcinoma (Keystone-001): Interim analysis of a prospective, single-arm, single-center, phase II trial. Ann Oncol (2021) 32:S1428–S9. doi: 10.1016/j.annonc.2021.10.218 PMC8908169 35280363

[43] Shen D Chen Q Wu J Li J Tao K Jiang Y . The safety and efficacy of neoadjuvant PD-1 inhibitor with chemotherapy for locally advanced esophageal squamous cell carcinoma. J Gastrointest Oncol (2021) 12(1):1–10. doi: 10.21037/jgo-20-599 33708420 PMC7944149

[44] Wang Z ed. Neoadjuvant camrelizumab combined with chemotherapy and apatinib for locally advanced thoracic esophageal squamous cell carcinoma (ESCC): A single-arm, open-label, phase ib study2021, in: ASCO Annual Meeting, JCO: American Society of Clinical Oncology (2021) 39(15_suppl):4047.

[45] Xing W Zhao L Zheng Y Liu B Liu X Li T . The sequence of chemotherapy and toripalimab might influence the efficacy of neoadjuvant chemoimmunotherapy in locally advanced esophageal squamous cell cancer–a phase II study. . Front Immunol (2021) 12:772450. doi: 10.3389/fimmu.2021.772450 34938292 PMC8685246

[46] Yamamoto S Kato K Daiko H Kojima T Hara H Abe T . FRONTiER: A feasibility trial of nivolumab with neoadjuvant CF or DCF therapy for locally advanced esophageal carcinoma(JCOG1804E)-the short-termresults of cohort a and b. J Clin Oncol (2021) 39(3 SUPPL):202. doi: 10.1200/JCO.2021.39.3_suppl.202 33332191

[47] Yan X Zhao J Lei J Duan H Ni Y Zhou Y . Tislelizumab combined with chemotherapy as neoadjuvant therapy for surgically resectable esophageal cancer (TD-NICE): A single arm, phase II study. Ann Oncol (2021) 32:S1442. doi: 10.1016/j.annonc.2021.10.163

[48] Yang P Zhou X Yang X Wang Y Sun T Feng S . Neoadjuvant camrelizumab plus chemotherapy in treating locally advanced esophageal squamous cell carcinoma patients: a pilot study. World J Surg Oncol (2021) 19(1):333. doi: 10.1186/s12957-021-02446-5 34809658 PMC8609728

[49] Yang W Xing X Yeung SJ Wang S Chen W Bao Y . Neoadjuvant programmed cell death 1 blockade combined with chemotherapy for resectable esophageal squamous cell carcinoma. J immunother Cancer (2022) 10(1):e003497. doi: 10.1136/jitc-2021-003497 35022193 PMC8756283

[50] Zhang G Hu Y Yang B Xu Q Li J Sun S . A single-centre, prospective, open-label, single-arm trial of toripalimab with nab-paclitaxel and s-1 as a neoadjuvant therapy for esophageal squamous cell carcinoma (ESCC). Ann Oncol (2020) 31:S722. doi: 10.1016/j.annonc.2020.08.1178

[51] Zhang X Yang G Su X Luo G Cai P Zheng Y . Neoadjuvant programmed death1 blockade plus chemotherapy in locally advanced esophageal squamous cell carcinoma. J Clin Oncol (2021) 39(15 SUPPL):e16076. doi: 10.1200/JCO.2021.39.15_suppl.e16076 PMC8421958 34532391

[52] Zhang Y Shen G Xu R Huang G Yang S Zheng Q . Real-world effectiveness and safety of camrelizumab-based neoadjuvant therapy in resectable esophageal cancer: Initial results of a prospective multicenter observational study. J Clin Oncol (2022) 40(4 SUPPL):250. doi: 10.1200/JCO.2022.40.4_suppl.250

[53] Zhang Z Hong Z-N Xie S Lin W Lin Y Zhu J . Neoadjuvant sintilimab plus chemotherapy for locally advanced esophageal squamous cell carcinoma: A single-arm, single-center, phase 2 trial (ESONICT-1). Ann Trans Med (2021) 9(21):1623. doi: 10.21037/atm-21-5381 PMC8640906 34926667

[54] Zhang Z Ye J Li H Du M Gu D Zhang J . 1378P a single-center, prospective, open-label, single-arm trial of sintilimab with paclitaxel and carboplatin as a neoadjuvant therapy for esophageal squamous carcinoma. Ann Oncol (2021) 32:S1042–S3. doi: 10.1016/j.annonc.2021.08.1487

[55] Wang F ed. Camrelizumab in combination with preoperative chemotherapy for locally advanced esophageal squamous cell carcinoma: A single-arm, open-label, phase II study2021, in: ASCO Annual Meeting, JCO: American Society of Clinical Oncology (2021) 39(3_suppl):222.

[56] Xu W Jiang Y Wang C Wu J Li J Hu Y . The efficacy and safety of neoadjuvant camrelizumab and chemotherapy for locally advanced thoracic esophageal squamous cell carcinoma. J Clin Oncol (2022) 40(4 SUPPL):278. doi: 10.1200/JCO.2022.40.4_suppl.278

[57] Hayashino Y Noguchi Y Fukui T . Systematic evaluation and comparison of statistical tests for publication bias. J Epidemiol (2005) 15(6):235–43. doi: 10.2188/jea.15.235 PMC7904376 16276033

[58] Zhao X Ren Y Hu Y Cui N Wang X Cui Y . Neoadjuvant chemotherapy versus neoadjuvant chemoradiotherapy for cancer of the esophagus or the gastroesophageal junction: A meta-analysis based on clinical trials. PloS One (2018) 13(8):e0202185. doi: 10.1371/journal.pone.0202185 30138325 PMC6107145

[59] Kelsen DP Ginsberg R Pajak TF Sheahan DG Gunderson L Mortimer J . Chemotherapy followed by surgery compared with surgery alone for localized esophageal cancer. N Engl J Med (1998) 339(27):1979–84. doi: 10.1056/NEJM199812313392704 9869669

[60] Kato K Ito Y Daiko H Ozawa S Ogata T Hara H . A randomized controlled phase III trial comparing two chemotherapy regimen and chemoradiotherapy regimen as neoadjuvant treatment for locally advanced esophageal cancer, JCOG1109 NExT study. J Clin Oncol (2022) 40(4_suppl):238. doi: 10.1200/JCO.2022.40.4_suppl.238

[61] Cunningham D Allum WH Stenning SP Thompson JN Van de Velde CJ Nicolson M . Perioperative chemotherapy versus surgery alone for resectable gastroesophageal cancer. N Engl J Med (2006) 355(1):11–20. doi: 10.1056/NEJMoa055531 16822992

[62] Mariette C Dahan L Mornex F Maillard E Thomas PA Meunier B . Surgery alone versus chemoradiotherapy followed by surgery for stage I and II esophageal cancer: final analysis of randomized controlled phase III trial FFCD 9901. J Clin Oncol (2014) 32(23):2416–22. doi: 10.1200/JCO.2013.53.6532 24982463

[63] Kelly RJ Ajani JA Kuzdzal J Zander T Van Cutsem E Piessen G . Adjuvant nivolumab in resected esophageal or gastroesophageal junction cancer. N Engl J Med (2021) 384(13):1191–203. doi: 10.1056/NEJMoa2032125 33789008

[64] Lagergren J Smyth E Cunningham D Lagergren P . Oesophageal cancer. Lancet (2017) 390(10110):2383–96. doi: 10.1016/S0140-6736(17)31462-9 28648400

[65] Sun H Yang W Luo J Lin H Zhou T Gong H . Safety and tolerability of neoadjuvant radiotherapy combined with anti-PD-1 antibody toripalimab for locally advanced, resectable esophageal squamous cell cancer: A prospective phase IB trial. Int J Radiat Oncol Biol Phys (2021) 111(3):S102–S3. doi: 10.1016/j.ijrobp.2021.07.238

[66] Altorki NK McGraw TE Borczuk AC Saxena A Port JL Stiles BM . Neoadjuvant durvalumab with or without stereotactic body radiotherapy in patients with early-stage non-small-cell lung cancer: a single-centre, randomised phase 2 trial. Lancet Oncol (2021) 22(6):824–35. doi: 10.1016/S1470-2045(21)00149-2 34015311

