# Supplementary material for: Efficacy and safety of neoadjuvant immunotherapy combined with chemoradiotherapy or chemotherapy in esophageal cancer: A systematic review and meta-analysis
Source: Front Immunol. 2023 Jan 24;14:1117448. doi: 10.3389/fimmu.2023.1117448 (PMC9902949; doi:10.3389/fimmu.2023.1117448)
Supplement: Supplementary file 1 [file Table_1.docx]

Supplementary Table 1. Scores of included studies by the methodological index for non-randomized studies (MINORS) index

| Author | A clearly stated aim | Inclusion of consecutive patients | Prospective collection of data | Endpoints appropriate to the aim of the study | Unbiased assessment of the study endpoint | Follow up period appropriate to the aim of the study | Loss to follow up less than 5 | Prospective calculation of the study size | An adequate control group | Contemporary groups | Baseline equivalence of groups | Adequate statistical analyses | Total |
| --- | --- | --- | --- | --- | --- | --- | --- | --- | --- | --- | --- | --- | --- |
| Cowzer, D., et al.(1) | 2 | 1 | 2 | 2 | 0 | 2 | 2 | 2 |  |  |  |  | 13 |
| Duan, H., et al.(2) | 2 | 1 | 2 | 2 | 0 | 2 | 2 | 2 |  |  |  |  | 13 |
| Gao, L. et al.(3) | 2 | 2 | 2 | 2 | 0 | 2 | 2 | 2 |  |  |  |  | 14 |
| Gu, Y., et al.(4) | 2 | 1 | 2 | 2 | 0 | 2 | 2 | 2 |  |  |  |  | 13 |
| Guo, J., et al.(5) | 2 | 1 | 2 | 2 | 0 | 2 | 2 | 2 |  |  |  |  | 13 |
| He, W., et al.(6) | 2 | 1 | 2 | 2 | 0 | 2 | 2 | 2 |  |  |  |  | 13 |
| Jiang, B., et al.(7) | 2 | 1 | 2 | 2 | 0 | 2 | 2 | 2 |  |  |  |  | 13 |
| Jiang, N., et al.(8) | 2 | 2 | 2 | 2 | 0 | 2 | 2 | 2 |  |  |  |  | 14 |
| Kelly, R. J., et al.(9) | 2 | 1 | 2 | 2 | 0 | 2 | 2 | 1 | 0 | 2 | 0 | 1 | 15 |
| Lee, S., et al. (10) | 2 | 1 | 2 | 2 | 0 | 2 | 2 | 2 |  |  |  |  | 13 |
| Li, C., et al.(11) | 2 | 2 | 2 | 2 | 0 | 2 | 2 | 2 |  |  |  |  | 14 |
| Li, K., et al.(12) | 2 | 1 | 2 | 2 | 0 | 2 | 2 | 2 |  |  |  |  | 13 |
| Li, Z., et al.(13) | 2 | 1 | 2 | 2 | 0 | 2 | 2 | 2 |  |  |  |  | 13 |
| Liu, D., et al. (14) | 2 | 1 | 2 | 2 | 0 | 2 | 2 | 2 |  |  |  |  | 13 |
| Liu, J.; Li, J., et al. (15) | 2 | 2 | 2 | 2 | 0 | 2 | 2 | 2 |  |  |  |  | 14 |
| Liu, J.; Yang, Y., et al.(16) | 2 | 1 | 2 | 2 | 0 | 2 | 2 | 2 |  |  |  |  | 13 |
| Ma, J., et al.(17) | 2 | 1 | 2 | 2 | 0 | 2 | 2 | 2 |  |  |  |  | 13 |
| Manish A. Shah, et al.(18) | 2 | 1 | 2 | 2 | 0 | 2 | 2 | 2 | 0 | 2 | 0 | 1 | 16 |
| Qi, W.X., et al.(19) | 2 | 1 | 2 | 2 | 0 | 2 | 2 | 2 |  |  |  |  | 13 |
| Shang, X., et al.(20) | 2 | 1 | 2 | 2 | 0 | 2 | 2 | 2 |  |  |  |  | 13 |
| Shen, D., et al.(21) | 2 | 1 | 2 | 2 | 0 | 2 | 2 | 2 |  |  |  |  | 13 |
| Uboha, NV., et al.(22) | 2 | 1 | 2 | 2 | 0 | 2 | 2 | 2 |  |  |  |  | 13 |
| van Den Ende, T., et al.(23) | 2 | 2 | 2 | 2 | 0 | 2 | 2 | 2 |  |  |  |  | 14 |
| Wang, F., et al.(24) | 2 | 1 | 2 | 2 | 0 | 2 | 2 | 2 |  |  |  |  | 13 |
| Wang, W., et al.(25) | 2 | 1 | 2 | 2 | 0 | 2 | 2 | 2 |  |  |  |  | 13 |
| Wang, Z., et al.(26) | 2 | 1 | 2 | 2 | 0 | 2 | 2 | 2 |  |  |  |  | 13 |
| Xing, W., et al.(27) | 2 | 1 | 2 | 2 | 0 | 2 | 2 | 1 | 0 | 2 | 2 | 2 | 18 |
| Xu, W., et al.(28) | 2 | 1 | 2 | 2 | 0 | 2 | 2 | 2 |  |  |  |  | 13 |
| Xu, X., et al.(29) | 2 | 1 | 2 | 2 | 0 | 2 | 2 | 2 |  |  |  |  | 13 |
| Yamamoto, S.; Matsuda, S., et al.(30, 31) | 2 | 1 | 2 | 2 | 0 | 2 | 2 | 1 | 0 | 2 | 1 | 0 | 15 |
| Yan, X., et al.(32) | 2 | 1 | 2 | 2 | 0 | 2 | 2 | 2 |  |  |  |  | 13 |
| Yang, P., et al.(33) | 2 | 1 | 2 | 2 | 0 | 2 | 2 | 2 |  |  |  |  | 13 |
| Yang, W., et al.(34) | 2 | 2 | 2 | 2 | 0 | 2 | 2 | 2 |  |  |  |  | 14 |
| Zhang, G., et al. (35) | 2 | 1 | 2 | 2 | 0 | 2 | 2 | 2 |  |  |  |  | 13 |
| Zhang, X.(36) | 2 | 1 | 2 | 2 | 0 | 2 | 2 | 2 |  |  |  |  | 13 |
| Zhang, Y., et al.(37) | 2 | 1 | 2 | 2 | 0 | 2 | 2 | 2 |  |  |  |  | 13 |
| Zhang, Z.; Hong, Z., et al.(38) | 2 | 2 | 2 | 2 | 0 | 2 | 2 | 2 |  |  |  |  | 14 |
| Zhang, Z.; Ye, J., et al.(39) | 2 | 2 | 2 | 2 | 0 | 2 | 2 | 2 |  |  |  |  | 14 |

References

1. Cowzer D, Wu AJ-C, Sihag S, Walch HS, Park BJ, Jones DR, et al. Durvalumab (D) and PET-directed chemoradiation (CRT) after induction FOLFOX for esophageal adenocarcinoma: Final results. Journal of Clinical Oncology. 2022;40(16_suppl):4029-.

2. Duan H, Wang T, Luo Z, Wang X, Liu H, Tong L, et al. A multicenter single-arm trial of sintilimab in combination with chemotherapy for neoadjuvant treatment of resectable esophageal cancer (SIN-ICE study). Annals of translational medicine. 2021;9(22).

3. Gao L, Lu J, Zhang P, Hong ZN, Kang M. Toripalimab combined with docetaxel and cisplatin neoadjuvant therapy for locally advanced esophageal squamous cell carcinoma: a single-center, single-arm clinical trial (ESONICT-2). Journal of Gastrointestinal Oncology. 2022;13(2):478-87.

4. Gu Y, Chen X, Wang D, Ding M, Xue L, Zhen F, et al. A study of neoadjuvant sintilimab combined with triplet chemotherapy of lipo-paclitaxel, cisplatin, and S-1 for resectable esophageal squamous cell carcinoma (ESCC). Annals of oncology : official journal of the European Society for Medical Oncology. 2020;31:S1307-S8.

5. Guo J, editor Neoadjuvant sintilimab combined with chemotherapy in patients with resectable esophageal squamous cell carcinoma (ESCC): Preliminary results from a phase II study2022; ASCO Annual Meeting: American Society of Clinical Oncology.

6. He W, Leng X, Mao T, Luo X, Zhou L, Yan J, et al. Toripalimab Plus Paclitaxel and Carboplatin as Neoadjuvant Therapy in Locally Advanced Resectable Esophageal Squamous Cell Carcinoma. The oncologist. 2022;27(1):e18-e28.

7. Jiang B, Yang X, Zhang J, Huang M. Abstract 5230: Neoadjuvant programmed cell death protein 1 inhibitors combined with chemotherapy in resectable esophageal squamous carcinoma: an open-label, single-arm study. Cancer Research. 2022;82(12_Supplement):5230-.

8. Jiang N, editor SCALE-1: Safety and efficacy of short course neoadjuvant chemo-radiotherapy plus toripalimab for locally advanced resectable squamous cell carcinoma of esophagus2022; ASCO Annual Meeting: American Society of Clinical Oncology.

9. Kelly RJ, Zaidi AH, van Liere Canzoniero J, Feliciano JL, Hales RK, Voong KR, et al. Multicenter phase II study of neoadjuvant nivolumab or nivolumab plus relatlimab (antiLAG3 antibody) plus chemoradiotherapy in stage II/III esophageal/gastroesophageal junction (E/GEJ) carcinoma. J Clin Oncol. 2022;40(4 SUPPL).

10. Lee S, Ahn BC, Park SY, Kim DJ, Lee CG, Cho J, et al. A phase II trial of preoperative chemoradiotherapy and pembrolizumab for locally advanced esophageal squamous cell carcinoma (ESCC). Annals of oncology : official journal of the European Society for Medical Oncology. 2019;30:v754.

11. Li C, Zhao S, Zheng Y, Han Y, Chen X, Cheng Z, et al. Preoperative pembrolizumab combined with chemoradiotherapy for oesophageal squamous cell carcinoma (PALACE-1). Eur J Cancer. 2021;144:232-41.

12. Li K, Yang X, Luo W, Ma Q, Wang Y, Xiong Y, et al. Toripalimab plus nab-paclitaxel and carboplatin as neoadjuvant therapy for patients with esophageal squamous cell carcinoma at clinical stage t2-t4/n0-n2/m0: A single-arm, single-center clinical study. Journal for ImmunoTherapy of Cancer. 2020;8(SUPPL 3):A253.

13. Li Z, editor A study of neoadjuvant sintilimab combined with chemotherapy TP for locally advanced esophageal squamous cell carcinoma (ESCC)2022; ASCO Annual Meeting: American Society of Clinical Oncology.

14. Liu D, Zhang Q, Zhu J, Qian T, Yin R, Fan Z, et al. Phase-II study of toripalimab combined with neoadjuvant chemotherapy for the treatment of resectable esophageal squamous cell carcinoma. J Clin Oncol. 2021;39(15 SUPPL).

15. Liu J, Li J, Lin W, Shao D, Depypere L, Zhang Z, et al. Neoadjuvant camrelizumab plus chemotherapy for resectable, locally advanced esophageal squamous cell carcinoma (NIC-ESCC2019): A multicenter, phase 2 study. Int J Cancer. 2022.

16. Liu J, Yang Y, Liu Z, Fu X, Cai X, Li H, et al. Multicenter, single-arm, phase II trial of camrelizumab and chemotherapy as neoadjuvant treatment for locally advanced esophageal squamous cell carcinoma. Journal for immunotherapy of cancer. 2022;10(3).

17. Ma J, Zhang J, Yang Y, Zheng D, Wang X, Liang H, et al. 65P Camrelizumab combined with paclitaxel and nedaplatin as neoadjuvant therapy for locally advanced esophageal squamous cell carcinoma (ESPRIT): A phase II, single-arm, exploratory research. Annals of oncology : official journal of the European Society for Medical Oncology. 2021;32:S1400.

18. Manish A. Shah KA, Syma Iqbal, Prashant Thakkar, Bryan J. Schneider, Rhonda Yantiss, Yiru Wu, Emma Futamura, Jeffrey L. Port, Cathy Spinelli, Sandipto Sarkar, Paul J. Christos, Gagandeep Brar, Doron Betel, Nicholas J. Sanfilippo, Nasser K. Altorki, editor Multicenter, randomized phase II study of neoadjuvant pembrolizumab plus chemotherapy and chemoradiotherapy in esophageal adenocarcinoma (EAC)2021; ASCO Annual Meeting: American Society of Clinical Oncology.

19. Qi WX, Zhao S, Li H, Chen J. Safety And Tolerability Of Neoadjuvant Chemoradiotherapy Combined With Pembrolizumab For Local Advanced, Resectable Esophageal Cancer: preliminary Results Of A Prospective Phase IB Trial. 2020;108(3):e576‐e7.

20. Shang X, Zhang C, Zhao G, Zhang W, Liu L, Duan X, et al. LBA3 Safety and efficacy of pembrolizumab combined with paclitaxel and cisplatin as a neoadjuvant treatment for locally advanced resectable (stage III) esophageal squamous cell carcinoma (Keystone-001): Interim analysis of a prospective, single-arm, single-center, phase II trial. Annals of oncology : official journal of the European Society for Medical Oncology. 2021;32:S1428-S9.

21. Shen D, Chen Q, Wu J, Li J, Tao K, Jiang Y. The safety and efficacy of neoadjuvant PD-1 inhibitor with chemotherapy for locally advanced esophageal squamous cell carcinoma. J Gastrointest Oncol. 2021;12(1):1–10.

22. Uboha NV, Eickhoff JC, Maloney JD, McCarthy D, DeCamp M, Deming DA, et al. Phase I/II trial of perioperative avelumab in combination with chemoradiation (CRT) in the treatment of stage II/III resectable esophageal and gastroesophageal junction (E/GEJ) cancer. Journal of Clinical Oncology. 2022;40(16_suppl):4034-.

23. van den Ende T, Clercq NCd, van Berge Henegouwen MI, Gisbertz SS, Geijsen ED, Verhoeven RHA, et al. Neoadjuvant chemoradiotherapy combined with atezolizumab for resectable esophageal adenocarcinoma: A single-arm phase ii feasibility trial (PERFECT). Clinical cancer research : an official journal of the American Association for Cancer Research. 2021;27(12):3351–9.

24. Wang F, editor Camrelizumab in combination with preoperative chemotherapy for locally advanced esophageal squamous cell carcinoma: A single-arm, open-label, phase II study2021; ASCO Annual Meeting: American Society of Clinical Oncology.

25. Wang W, editor Neoadjuvant pembrolizumab plus chemotherapy for resectable locally advanced esophageal squamous cell carcinoma (ESCC): Interim results2022; ASCO Annual Meeting: American Society of Clinical Oncology.

26. Wang Z, editor Neoadjuvant camrelizumab combined with chemotherapy and apatinib for locally advanced thoracic esophageal squamous cell carcinoma (ESCC): A single-arm, open-label, phase Ib study2021; ASCO Annual Meeting: American Society of Clinical Oncology.

27. Xing W, Zhao L, Zheng Y, Liu B, Liu X, Li T, et al. The Sequence of Chemotherapy and Toripalimab Might Influence the Efficacy of Neoadjuvant Chemoimmunotherapy in Locally Advanced Esophageal Squamous Cell Cancer—A Phase II Study. Front Immunol. 2021;12.

28. Xu W, Jiang Y, Wang C, Wu J, Li J, Hu Y, et al. The efficacy and safety of neoadjuvant camrelizumab and chemotherapy for locally advanced thoracic esophageal squamous cell carcinoma. J Clin Oncol. 2022;40(4 SUPPL).

29. Xu X, editor Neoadjuvant chemoradiotherapy combined with perioperative toripalimab in locally advanced esophageal cancer2022; ASCO Annual Meeting: American Society of Clinical Oncology.

30. Yamamoto S, Kato K, Daiko H, Kojima T, Hara H, Abe T, et al. FRONTiER: A feasibility trial of nivolumab with neoadjuvant CF or DCF therapy for locally advanced esophageal carcinoma(JCOG1804E)-The short-termresults of cohort A and B. J Clin Oncol. 2021;39(3 SUPPL).

31. Matsuda S, Yamamoto S, Kato K, Daiko H, Kojima T, Hara H, et al. FRONTiER: A feasibility trial of nivolumab with neoadjuvant CF or DCF, FLOT therapy for locally advanced esophageal carcinoma (JCOG1804E)-Short-term results for cohorts C and D. J Clin Oncol. 2022;40(4 SUPPL).

32. Yan X, Zhao J, Lei J, Duan H, Ni Y, Zhou Y, et al. Tislelizumab combined with chemotherapy as neoadjuvant therapy for surgically resectable esophageal cancer (TD-NICE): A single arm, phase II study. Annals of oncology : official journal of the European Society for Medical Oncology. 2021;32:S1442.

33. Yang P, Zhou X, Yang X, Wang Y, Sun T, Feng S, et al. Neoadjuvant camrelizumab plus chemotherapy in treating locally advanced esophageal squamous cell carcinoma patients: a pilot study. World J Surg Oncol. 2021;19(1).

34. Yang W, Xing X, Yeung SJ, Wang S, Chen W, Bao Y, et al. Neoadjuvant programmed cell death 1 blockade combined with chemotherapy for resectable esophageal squamous cell carcinoma. Journal for immunotherapy of cancer. 2022;10(1).

35. Zhang G, Hu Y, Yang B, Xu Q, Li J, Sun S, et al. A single-centre, prospective, open-label, single-arm trial of toripalimab with nab-paclitaxel and S-1 as a neoadjuvant therapy for esophageal squamous cell carcinoma (ESCC). Annals of oncology : official journal of the European Society for Medical Oncology. 2020;31:S722.

36. Zhang X, Yang G, Su X, Luo G, Cai P, Zheng Y, et al. Neoadjuvant programmed death1 blockade plus chemotherapy in locally advanced esophageal squamous cell carcinoma. J Clin Oncol. 2021;39(15 SUPPL).

37. Zhang Y, Shen G, Xu R, Huang G, Yang S, Zheng Q, et al. Real-world effectiveness and safety of camrelizumab-based neoadjuvant therapy in resectable esophageal cancer: Initial results of a prospective multicenter observational study. J Clin Oncol. 2022;40(4 SUPPL).

38. Zhang Z, Hong Z-N, Xie S, Lin W, Lin Y, Zhu J, et al. Neoadjuvant sintilimab plus chemotherapy for locally advanced esophageal squamous cell carcinoma: A single-arm, single-center, phase 2 trial (ESONICT-1). Annals of translational medicine. 2021;9(21).

39. Zhang Z, Ye J, Li H, Du M, Gu D, Zhang J, et al. 1378P A single-center, prospective, open-label, single-arm trial of sintilimab with paclitaxel and carboplatin as a neoadjuvant therapy for esophageal squamous carcinoma. Annals of oncology : official journal of the European Society for Medical Oncology. 2021;32:S1042-S3.
