# Supplementary material for: Efficacy and safety of neoadjuvant immunotherapy combined with chemoradiotherapy or chemotherapy in esophageal cancer: A systematic review and meta-analysis
Source: Front Immunol. 2023 Jan 24;14:1117448. doi: 10.3389/fimmu.2023.1117448 (PMC9902949; doi:10.3389/fimmu.2023.1117448)
Supplement: Supplementary file 2 [file Table_2.docx]

Supplementary Table 2. Publication bias test of outcomes.

| Outcomes | Begg’s test (p-value) | Egger’s test (p-value) |
| --- | --- | --- |
| pCR | 0.247 | 0.416 |
| MPR | 0.269 | 0.809 |
| R0 resection rate | 0.030 | 0.372 |
| Incidence of ≥grade 3 TRAEs | 0.043 | 0.004 |
| NTCR | 0.021 | < 0.001 |
| Surgical resection rate | < 0.001 | < 0.001 |
| Surgical delay rate | 0.005 | 0.093 |

pCR, pathological complete response; MPR, major complete response; NTCR, neoadjuvant therapy completion rate
