# Supplementary material for: Efficacy and safety of neoadjuvant immunotherapy combined with chemoradiotherapy or chemotherapy in esophageal cancer: A systematic review and meta-analysis
Source: Front Immunol. 2023 Jan 24;14:1117448. doi: 10.3389/fimmu.2023.1117448 (PMC9902949; doi:10.3389/fimmu.2023.1117448)
Supplement: Supplementary file 3 [file DataSheet_1.docx]

Supplementary Material

Efficacy and safety of neoadjuvant immunotherapy combined with chemoradiotherapy or chemotherapy in esophageal cancer: a systematic review and meta-analysis

**Yunsong Liu, MD , Yongxing Bao, MD, Xu Yang, MD, Shuang Sun, MD, Meng Yuan, MD, Zeliang Ma, MD, Wanting Zhang, MD, Yirui Zhai, MD, Yang Wang, MSc, Yu Men, MD, Jianjun Qin, MD, Liyan Xue, MD, Jun Wang, MD, Zhouguang Hui, MD***

*** Correspondence:**

Zhouguang Hui, MD.
drhuizg@163.com

# Supplementary Data

1. Search strategy

(1).Pubmed

("Esophageal Neoplasms"[Mesh] OR ((esophageal[tiab] OR esophagus[tiab] OR oesophageal[tiab] OR oesophagus[tiab] OR gastroesophageal[tiab] OR oesophagogastric[tiab] OR esophagogastric[tiab]) AND (cancer[tiab] OR cancers[tiab] OR tumor[tiab] OR tumour[tiab] OR tumors[tiab] OR tumours[tiab] OR neoplasm[tiab] or neoplasms[tiab] OR malignancy[tiab] OR malignancies[tiab] OR adenocarcinoma[tiab] OR adenocarcinomas[tiab] OR carcinoma[tiab] OR carcinomas[tiab])))AND("Neoadjuvant Therapy"[Mesh] OR Neoadjuvant Therapies[tiab] OR Therapy, Neoadjuvant[tiab] OR Neoadjuvant Treatment[tiab] OR Neoadjuvant Treatments[tiab] OR Treatment, Neoadjuvant[tiab] OR Neoadjuvant Radiotherapy[tiab] OR Neoadjuvant Radiotherapies[tiab] OR Radiotherapy, Neoadjuvant[tiab] OR Neoadjuvant Radiation Treatment[tiab] OR Neoadjuvant Radiation Treatments[tiab] OR Radiation Treatment, Neoadjuvant[tiab] OR Treatment, Neoadjuvant Radiation[tiab] OR Neoadjuvant Radiation Therapy[tiab] OR Neoadjuvant Radiation Therapies[tiab] OR Radiation Therapy, Neoadjuvant[tiab] OR Therapy, Neoadjuvant Radiation[tiab] OR Neoadjuvant Radiation[tiab] OR Neoadjuvant Radiations[tiab] OR Radiation, Neoadjuvant[tiab] OR Neoadjuvant Systemic Therapy[tiab] OR Neoadjuvant Systemic Therapies[tiab] OR Systemic Therapy, Neoadjuvant[tiab] OR Therapy, Neoadjuvant Systemic[tiab] OR Neoadjuvant Systemic Treatment[tiab] OR Neoadjuvant Systemic Treatments[tiab] OR Systemic Treatment, Neoadjuvant[tiab] OR Treatment, Neoadjuvant Systemic[tiab] OR Neoadjuvant Chemotherapy[tiab] OR Chemotherapy, Neoadjuvant[tiab] OR Neoadjuvant Chemotherapies[tiab] OR Neoadjuvant Chemotherapy Treatment[tiab] OR Chemotherapy Treatment, Neoadjuvant[tiab] OR Neoadjuvant Chemotherapy Treatments[tiab] OR Treatment, Neoadjuvant Chemotherapy[tiab] OR Neoadjuvant Chemoradiotherapy[tiab] OR Chemoradiotherapy, Neoadjuvant[tiab] OR Neoadjuvant Chemoradiotherapies[tiab] OR Neoadjuvant Chemoradiation Therapy[tiab] OR Chemoradiation Therapy, Neoadjuvant[tiab] OR Neoadjuvant Chemoradiation Therapies[tiab] OR Therapy, Neoadjuvant Chemoradiation[tiab] OR Neoadjuvant Chemoradiation Treatment[tiab] OR Chemoradiation Treatment, Neoadjuvant[tiab] OR Neoadjuvant Chemoradiation Treatments[tiab] OR Treatment, Neoadjuvant Chemoradiation[tiab] OR Neoadjuvant Chemoradiation[tiab] OR Chemoradiation, Neoadjuvant[tiab] OR Neoadjuvant Chemoradiations[tiab] OR pre-surgical[tiab] OR presurgical[tiab] OR pre-operative[tiab] OR preoperative[tiab] OR neoadjuvant[tiab] ) AND (("Immunotherapy"[Mesh] OR immunotherapy[tiab] OR Immunotherapies[tiab] ) OR ("Ipilimumab"[Mesh] OR Anti-CTLA-4 MAb Ipilimumab[tiab] OR Anti CTLA 4 MAb Ipilimumab[tiab] OR Ipilimumab, Anti-CTLA-4 Mab[tiab] OR Yervoy[tiab] OR MDX 010[tiab] OR MDX010[tiab] OR MDX-010[tiab] OR MDX-CTLA-4[tiab] OR MDX CTLA 4[tiab] ) OR (pd-1 blockade[tiab] OR pd-1 checkpoints inhibitors[tiab] OR PD-1[tiab] OR "Nivolumab"[Mesh] OR Opdivo[tiab] OR ONO-4538[tiab] OR ONO 4538[tiab] OR ONO4538[tiab] OR MDX OR 1106[tiab] OR MDX 1106[tiab] OR MDX1106[tiab] OR BMS-936558[tiab] OR BMS 936558[tiab] OR BMS936558[tiab] OR pembrolizumab[tiab] OR lambrolizumab[tiab] OR Keytruda[tiab] OR MK-3475[tiab] OR sintilimab[tiab] OR IBI 308[tiab] OR IBI308[tiab] OR IBI-308[tiab] OR camrelizumab[tiab] OR SHR-1210[tiab] OR SHR 1210[tiab] OR Cemiplimab[tiab] OR REGN2810[tiab] OR toripalimab[tiab] OR Tislelizumab[tiab] OR BGB-A317[tiab] ) OR (PD-L1 blockade[tiab] OR PD-L1 checkpoints inhabitors[tiab] OR PD-L1[tiab] OR Atezolizumab[tiab] OR MPDL3280A[tiab] OR Tecentriq[tiab] OR RG7446[tiab] OR RG-7446[tiab] OR durvalumab[tiab] OR MEDI4736[tiab] OR MEDI-4736[tiab] OR Imfinzi[tiab] OR avelumab[tiab] OR MSB-0010682[tiab] OR MSB0010682[tiab] OR bavencio[tiab] OR MSB0010718C[tiab] OR MSB-0010718C[tiab] OR Sugemalimab[tiab]))

(2).Embase

('esophagus tumor'/exp OR 'esophageal':ab,ti OR 'esophagus':ab,ti OR 'oesophageal':ab,ti OR 'oesophagus':ab,ti OR 'gastroesophageal':ab,ti OR 'oesophagogastric':ab,ti OR 'esophagogastric':ab,ti) AND ('cancer':ab,ti OR 'cancers':ab,ti OR 'tumor':ab,ti OR 'tumour':ab,ti OR 'tumors':ab,ti OR 'tumours':ab,ti OR 'neoplasm':ab,ti OR 'neoplasms':ab,ti OR 'malignancy':ab,ti OR 'malignancies':ab,ti OR 'adenocarcinoma':ab,ti OR 'adenocarcinomas':ab,ti OR 'carcinoma':ab,ti OR 'carcinomas':ab,ti) AND ('neoadjuvant therapy'/exp OR 'neoadjuvant therapy':ab,ti OR 'neoadjuvant therapies':ab,ti OR 'therapies, neoadjuvant':ab,ti OR 'therapy, neoadjuvant':ab,ti OR 'neoadjuvant treatment':ab,ti OR 'neoadjuvant treatments':ab,ti OR 'treatment, neoadjuvant':ab,ti OR 'treatments, neoadjuvant':ab,ti OR 'pre-surgical':ab,ti OR 'presurgical':ab,ti OR 'pre-operative':ab,ti OR 'preoperative':ab,ti OR 'neoadjuvant':ab,ti) AND ('immunotherapy'/exp OR 'immunotherapies':ab,ti OR 'ipilimumab':ab,ti OR 'anti-ctla-4 mab ipilimumab':ab,ti OR 'anti ctla 4 mab ipilimumab':ab,ti OR 'ipilimumab, anti-ctla-4 mab':ab,ti OR 'yervoy':ab,ti OR 'mdx 010':ab,ti OR 'mdx010':ab,ti OR 'mdx-010':ab,ti OR 'mdx-ctla-4':ab,ti OR 'mdx ctla 4':ab,ti OR 'pd-1 blockade':ab,ti OR 'pd-1 checkpoints inhibitors':ab,ti OR 'pd-1':ab,ti OR 'nivolumab':ab,ti OR 'opdivo':ab,ti OR 'ono-4538':ab,ti OR 'ono 4538':ab,ti OR 'ono4538':ab,ti OR 'mdx':ab,ti OR '1106':ab,ti OR 'mdx 1106':ab,ti OR 'mdx1106':ab,ti OR 'bms-936558':ab,ti OR 'bms 936558':ab,ti OR 'bms936558':ab,ti OR 'pembrolizumab':ab,ti OR 'lambrolizumab':ab,ti OR 'keytruda':ab,ti OR 'mk-3475':ab,ti OR 'sintilimab':ab,ti OR 'ibi 308':ab,ti OR 'ibi308':ab,ti OR 'ibi-308':ab,ti OR 'camrelizumab':ab,ti OR 'shr-1210':ab,ti OR 'shr 1210':ab,ti OR 'cemiplimab':ab,ti OR 'regn2810':ab,ti OR 'toripalimab':ab,ti OR 'tislelizumab':ab,ti OR 'bgb-a317':ab,ti OR 'pd-l1 blockade':ab,ti OR 'pd-l1 checkpoints inhabitors':ab,ti OR 'pd-l1':ab,ti OR 'atezolizumab':ab,ti OR 'mpdl3280a':ab,ti OR 'tecentriq':ab,ti OR 'rg7446':ab,ti OR 'rg-7446':ab,ti OR 'durvalumab':ab,ti OR 'medi4736':ab,ti OR 'medi-4736':ab,ti OR 'imfinzi':ab,ti OR 'avelumab':ab,ti OR 'msb-0010682':ab,ti OR 'msb0010682':ab,ti OR 'bavencio':ab,ti OR 'msb0010718c':ab,ti OR 'msb-0010718c':ab,ti OR 'sugemalimab':ab,ti)

(3).Cochrane

((MeSH descriptor: [Esophageal Neoplasms] explode all trees)OR(((esophageal OR esophagus OR oesophageal OR oesophagus OR gastroesophageal OR oesophagogastric OR esophagogastric) AND (cancer OR cancers OR tumor OR tumour OR tumors OR tumours OR neoplasm or neoplasms OR malignancy OR malignancies OR adenocarcinoma OR adenocarcinomas OR carcinoma OR carcinomas)):ti,ab,kw))AND((MeSH descriptor: [Neoadjuvant Therapy] explode all trees)OR((Neoadjuvant Therapy OR Neoadjuvant Therapies OR Therapies, Neoadjuvant OR Therapy, Neoadjuvant OR Neoadjuvant Treatment OR Neoadjuvant Treatments OR Treatment, Neoadjuvant OR Treatments, Neoadjuvant OR pre-surgical OR presurgical OR pre-operative OR preoperative OR neoadjuvant):ti,ab,kw))AND((MeSH descriptor: [Immunotherapy] explode all trees)OR(

(Immunotherapies):ti,ab,kw))AND(((pd-1 blockade OR pd-1 checkpoints inhibitors OR PD-1 OR Nivolumab OR Opdivo OR ONO-4538 OR ONO 4538 OR ONO4538 OR MDX OR 1106 OR MDX 1106 OR MDX1106 OR BMS-936558 OR BMS 936558 OR BMS936558 OR pembrolizumab OR lambrolizumab OR Keytruda OR MK-3475 OR sintilimab OR IBI 308 OR IBI308 OR IBI-308 OR camrelizumab OR SHR-1210 OR SHR 1210 OR Cemiplimab OR REGN2810 OR toripalimab OR Tislelizumab OR BGB-A317):ti,ab,kw OR((PD-L1 blockade OR PD-L1 checkpoints inhabitors OR PD-L1 OR Atezolizumab OR MPDL3280A OR Tecentriq OR RG7446 OR RG-7446 OR durvalumab OR MEDI4736 OR MEDI-4736 OR Imfinzi OR avelumab OR MSB-0010682 OR MSB0010682 OR bavencio OR MSB0010718C OR MSB-0010718C OR Sugemalimab):ti,ab,kw))


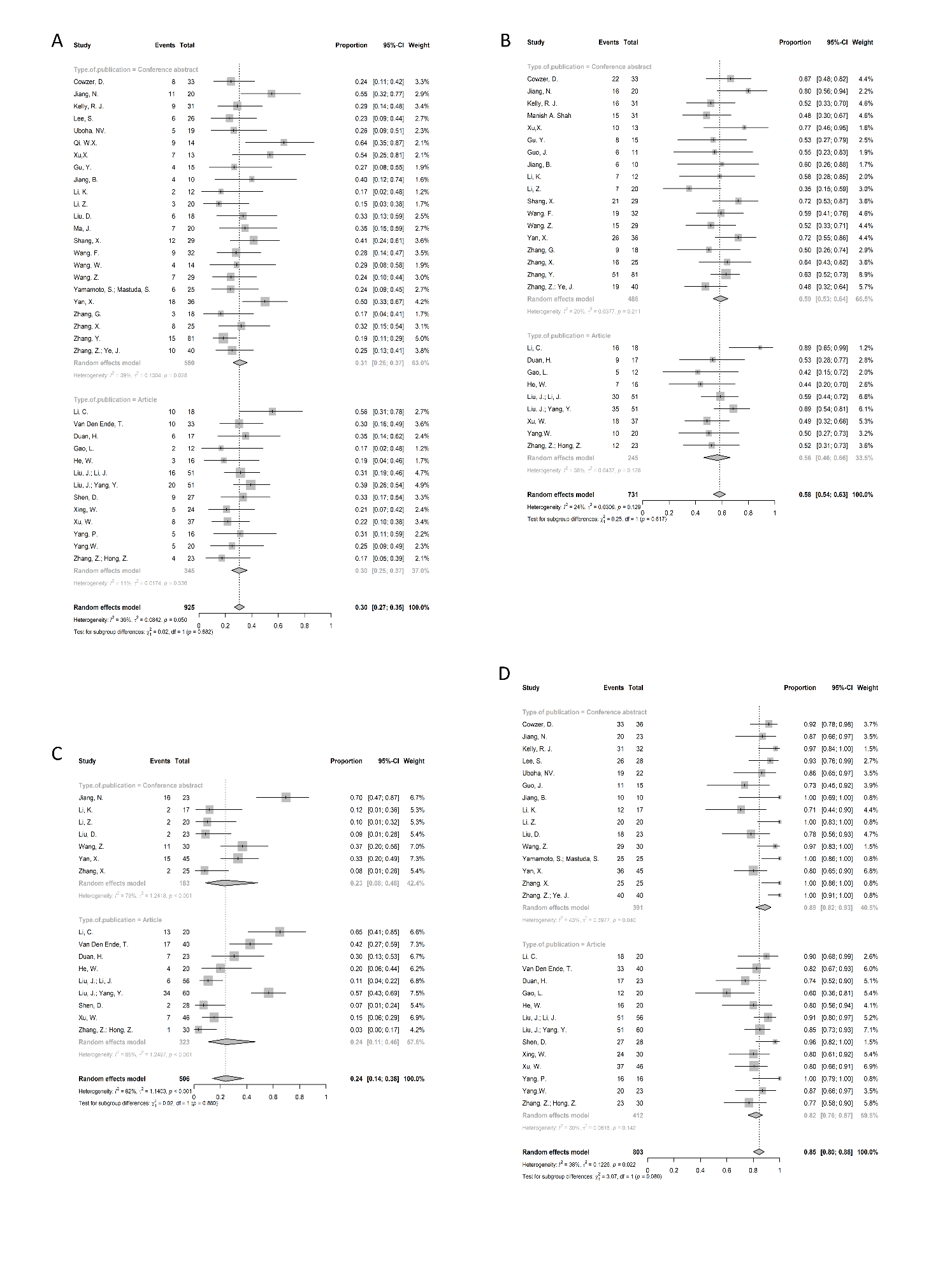


eFigure.1. Forest plots for subgroup analysis by types of publication

(A) Pathological complete response (pCR), (B) Major pathological response (MPR), (C) Incidence of ≥grade 3 TRAEs, and (D) Surgical resection rate.


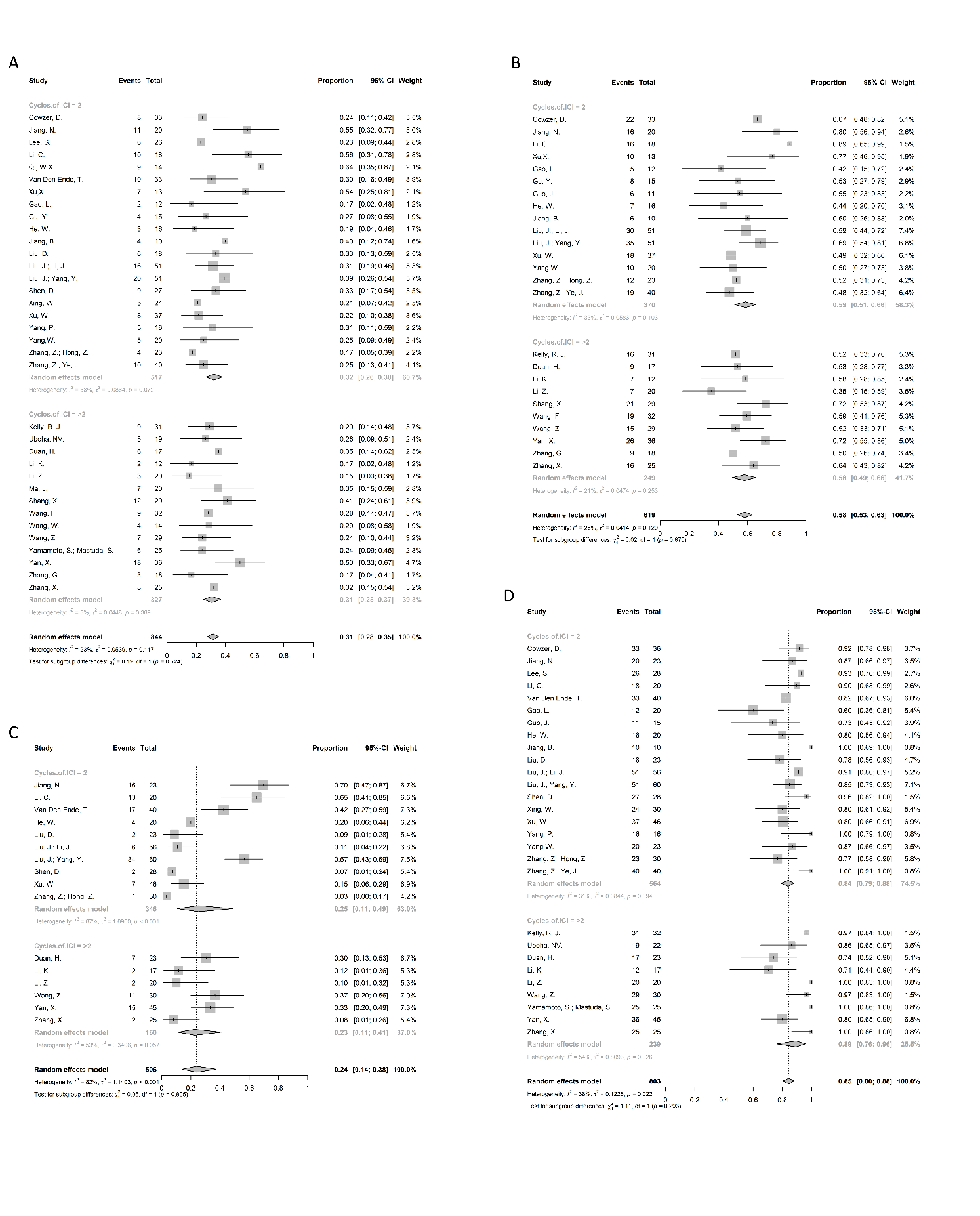


eFigure.2. Forest plots for subgroup analysis by cycles of ICI

(A) Pathological complete response (pCR), (B) Major pathological response (MPR), (C) Incidence of ≥grade 3 TRAEs, and (D) Surgical resection rate.


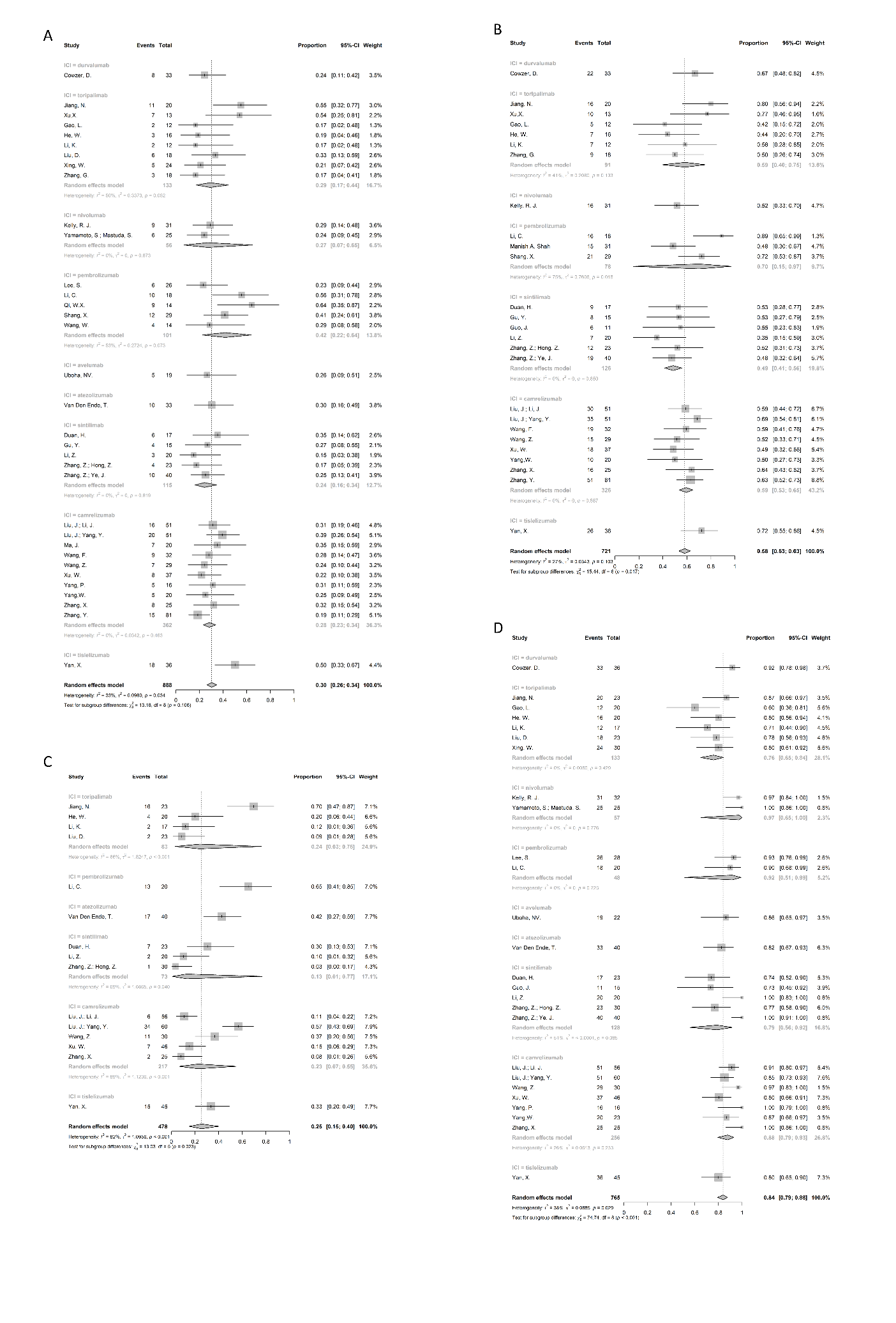


eFigure.3. Forest plots for subgroup analysis by types of ICI

(A) Pathological complete response (pCR), (B) Major pathological response (MPR), (C) Incidence of ≥grade 3 TRAEs, and (D) Surgical resection rate.

eTable.1. Certainty assessment by GRADE approach

| Outcomes | No. of participants (studies) | Risk of bias | Inconsistency | Indirectness | Imprecision | Publication bias | Overall certainty of evidence |
| --- | --- | --- | --- | --- | --- | --- | --- |
| pCR | 925 (36) | not serious | not serious | not serious | not serious | unlikely | low |
| MPR | 731 (27) | not serious | not serious | not serious | not serious | unlikely | low |
| R0 resection rate | 698 (27) | not serious | not serious | not serious | not serious | unlikely | low |
| Incidence of ≥grade 3 TRAEs | 506 (16) | not serious | serious | not serious | serious | strongly suspected | very low |
| NTCR | 444 (14) | not serious | not serious | not serious | not serious | strongly suspected | very low |
| Surgical resection rate | 803 (28) | not serious | not serious | not serious | not serious | strongly suspected | very low |
| Surgical delay rate | 426 (16) | not serious | not serious | not serious | serious | unlikely | very low |

Abbreviation: pCR, pathological complete response; MPR, major complete response; NTCR, neoadjuvant therapy completion rate

**
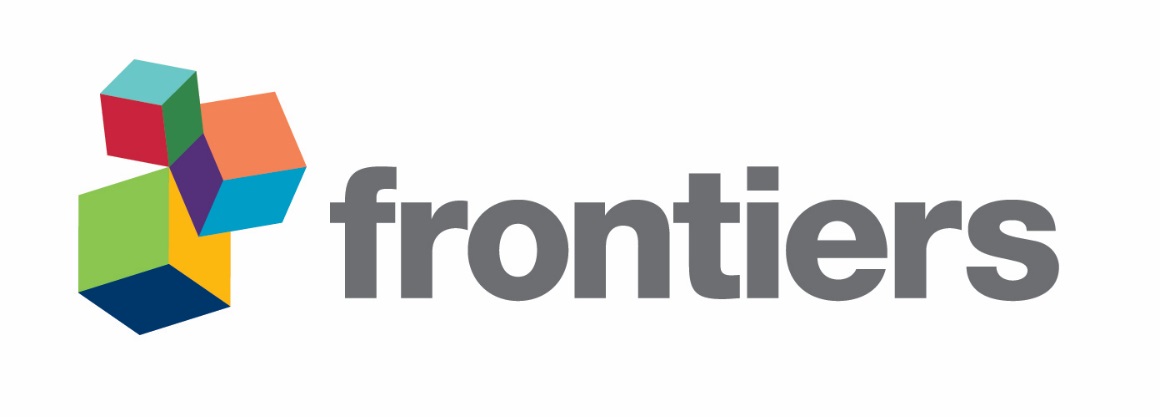
**
